# In Vitro and In Vivo Pipeline for Validation of Disease-Modifying Effects of Systems Biology-Derived Network Treatments for Traumatic Brain Injury—Lessons Learned

**DOI:** 10.3390/ijms20215395

**Published:** 2019-10-29

**Authors:** Anssi Lipponen, Teemu Natunen, Mika Hujo, Robert Ciszek, Elina Hämäläinen, Jussi Tohka, Mikko Hiltunen, Jussi Paananen, David Poulsen, Emilia Kansanen, Xavier Ekolle Ndode-Ekane, Anna-Liisa Levonen, Asla Pitkänen

**Affiliations:** 1 A. I. Virtanen Institute for Molecular Sciences, University of Eastern Finland, PO Box 1627, FIN-70211 Kuopio, Finland; anssi.lipponen@uef.fi (A.L.); robert.ciszek@uef.fi (R.C.); elina.hamalainen@uef.fi (E.H.); jussi.tohka@uef.fi (J.T.); emilia.kansanen@uef.fi (E.K.); xavier.ekollendode-ekane@uef.fi (X.E.N.-E.); anna-liisa.levonen@uef.fi (A.-L.L.); 2 Institute of Biomedicine, University of Eastern Finland, PO Box 1627, FIN-70211 Kuopio, Finland; teemu.natunen@uef.fi (T.N.); mikko.hiltunen@uef.fi (M.H.); jussi.paananen@uef.fi (J.P.); 3 School of Computing, University of Eastern Finland, PO Box 1627, FIN-70211 Kuopio, Finland; mika.hujo@uef.fi; 4 Bioinformatics Center, University of Eastern Finland, PO Box 1627, FIN-70211 Kuopio, Finland; 5 Jacobs School of Medicine and Biomedical Sciences, University of Buffalo, 875 Ellicott St, 6071 CTRC, Buffalo, NY 14203, USA; davidpou@buffalo.edu

**Keywords:** adverse event, common data element, LINCS analysis, machine-learning, traumatic brain injury, neuroinflammation, neuroprotection

## Abstract

We developed a pipeline for the discovery of transcriptomics-derived disease-modifying therapies and used it to validate treatments in vitro and in vivo that could be repurposed for TBI treatment. Desmethylclomipramine, ionomycin, sirolimus and trimipramine, identified by in silico LINCS analysis as candidate treatments modulating the TBI-induced transcriptomics networks, were tested in neuron-BV2 microglial co-cultures, using tumour necrosis factor α as a monitoring biomarker for neuroinflammation, nitrite for nitric oxide-mediated neurotoxicity and microtubule associated protein 2-based immunostaining for neuronal survival. Based on (a) therapeutic time window in silico, (b) blood-brain barrier penetration and water solubility, (c) anti-inflammatory and neuroprotective effects in vitro (*p* < 0.05) and (d) target engagement of Nrf2 target genes (*p* < 0.05), desmethylclomipramine was validated in a lateral fluid-percussion model of TBI in rats. Despite the favourable in silico and in vitro outcomes, in vivo assessment of clomipramine, which metabolizes to desmethylclomipramine, failed to demonstrate favourable effects on motor and memory tests. In fact, clomipramine treatment worsened the composite neuroscore (*p* < 0.05). Weight loss (*p* < 0.05) and prolonged upregulation of plasma cytokines (*p* < 0.05) may have contributed to the worsened somatomotor outcome. Our pipeline provides a rational stepwise procedure for evaluating favourable and unfavourable effects of systems-biology discovered compounds that modulate post-TBI transcriptomics.

## 1. Introduction

Traumatic brain injury (TBI) affects approximately 2.6 million people in Europe and the USA annually, resulting in chronic disabilities in more than 40% of TBI patients [1,2]. TBI triggers a cascade of parallel and sequential molecular changes in the injured brain that underlie the evolution of post-TBI secondary pathologies, including apoptosis, inflammation and oxidative stress [3,4]. Various approaches targeting different secondary pathologies have been tested in proof-of-concept preclinical trials, with over 100 demonstrating some positive effect on post-TBI outcome [5,6,7,8,9]. Translation of preclinical findings to the clinic has been inefficient, however and recovery-enhancing treatments for TBI are still lacking in clinical practice.

The majority of drug-discovery studies for TBI have used a hypothesis-driven “single target/single drug approach.” Non-hypothesis driven in silico approaches, however, were recently demonstrated to present a viable strategy in drug discovery for complex brain diseases such as Alzheimer disease, Parkinson disease, Huntington disease, schizophrenia and epilepsy [10,11,12,13,14,15,16]. The in silico network approach applies molecular omics of tissue pathology in drug discovery, allowing for unbiased identification of pharmacotherapies that target more than one disease-activated molecular network [17,18,19,20,21]. These studies have screened large compound libraries, such as the Connectivity Map (CMap) and Library of Integrated Network-Based Cellular Signatures (LINCS) [11,22], to identify not only novel compounds but also compounds that can be repurposed to hasten clinical implementation.

Our previous in silico studies revealed desmethylclomipramine, ionomycin, sirolimus and trimipramine as modulators of chronic post-TBI transcriptomics [22,23]. Using in silico analysis, however, it is difficult to determine whether the modulation of gene expression results in favourable or unfavourable cellular and functional outcomes. Therefore, we developed a stepwise in vitro and in vivo pipeline to systematically screen in silico-predicted TBI treatments using monitoring biomarkers for neuroinflammation, nitric oxide –mediated neurotoxicity and neuronal survival. In vivo data were collected using common data elements and stored in a RedCAP database [24]. Based on go/no-go decisions at each step, the disease-modifying effects of clomipramine, which is rapidly metabolized to its active metabolite, desmethylclomipramine, were tested in an in vivo proof-of-concept trial in a rat lateral fluid-percussion model of TBI. The data show the usefulness of the pipeline and emphasizes the need for systematic monitoring of both therapeutic effects and adverse events.

## 2. Results

**Figure 1:**
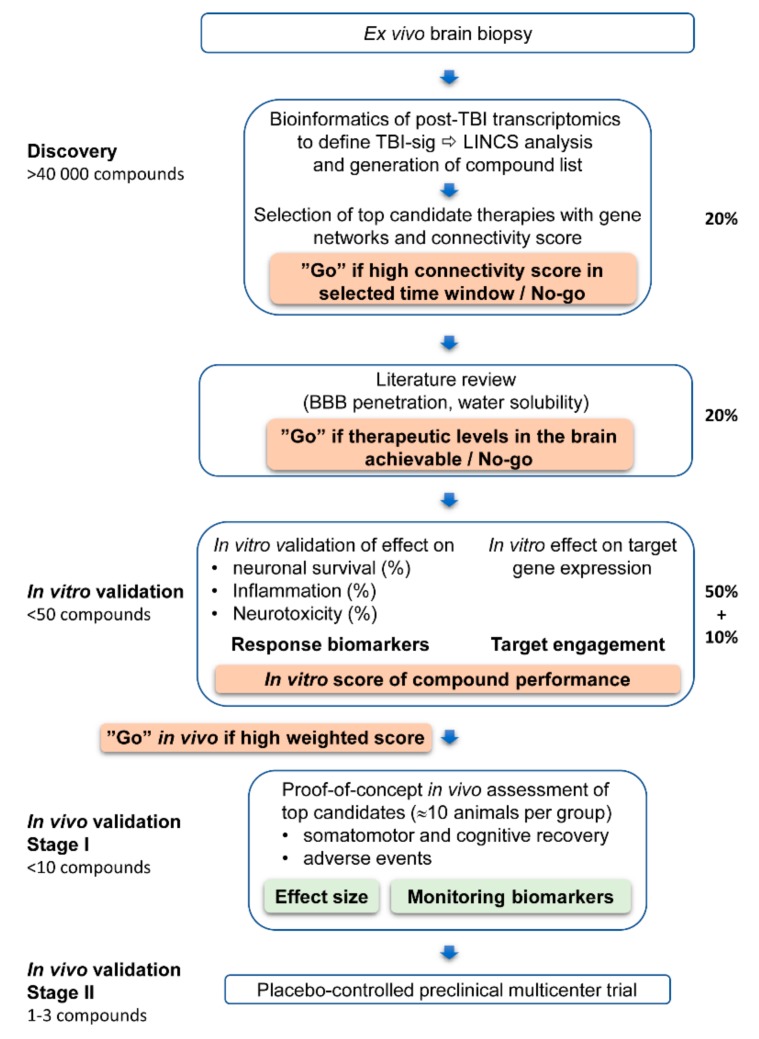
Overview of the pipeline for in vitro and in vivo validation of in silico-derived candidate treatments for traumatic brain injury (TBI). Weighted scoring scheme is presented in Section 2.4 and Section 4.2.5. Percentage on the right indicates the weight of each component in the total score. On the left we indicate our rough estimate on filtering-out of the compounds during the procedure within a 3-y time frame based on the data obtained. Discovery phase: Bioinformatics of mRNA-sequencing (seq) data of tissue sampled from the brain region of interest. For example, we sampled the perilesional cortex, which is critical for epileptogenesis after TBI. The data are used to generate a TBI transcriptomics signature (TBI-sig) that is then compared with the compound-induced transcriptomics signature (compound-sig), for example, in the iLINCS database, which includes over 40,000 compound-sig (http://www.ilincs.org). A list of compounds with concordance scores is generated and the ones with the highest absolute values of concordance scores will be further evaluated by manual literature search. In vitro validation phase: A limited number of compounds with a high concordance score and favourable pharmacokinetic and toxicity profiles will be tested in in vitro models. For example, we tested desmethylclomipramine, ionomycin, sirolimus and trimipramine. We used neuronal-BV2 microglial cultures with lipopolysaccharide/interferon gamma (LPS/IFNγ) -induced inflammation. We assessed the effect of test compounds (% of untreated control) on neuroinflammation using tumour necrosis factor alpha (TNFα), nitric oxide-mediated neurotoxicity using nitrite levels and neuronal viability using microtubule-associated protein 2 (MAP2) as monitoring biomarkers. Also, target engagement of the test compounds (down-regulation vs. up-regulation) will be tested in this phase. Compounds with target engagement within the desired time window and favourable effects on monitoring biomarkers will proceed to the next step. The weighted scoring shown in Tables 4 and 5 guides the Go/No-go decision. In vivo validation: The first stage in in vivo validation includes a proof-of-concept demonstration of favourable effects on performance in behavioural and cognitive tests. For example, we expected the treatment to improve performance in neuroscore, beam-walking and Morris water-maze tests to a level no different from that in the Vehicle-TBI group at the number of animals used (~10 per group). Also, we monitored adverse events (temperature, weight, overall appearance). We also measured plasma cytokines as monitoring biomarkers for drug effects and analysed the data using mathematical modelling and machine learning. Based on the data obtained, we did not proceed to the second stage of in vivo validation to reproduce the data with a higher number animals, which would be necessary to address subtle effects in a subpopulation of animals. This stage could also include more specific outcome measures such as the effect of compounds on post-impact epileptiform activity, which would require video-electroencephalogram recording.

The screening pipeline is outlined in Figure 1.

Selection of in silico discovered compounds to validation. Our earlier in silico analysis identified antidepressants, anti-cancer drugs and bioactive compounds as modifiers of post-TBI transcriptomics changes [22,23]. Four of these compounds were chosen for the present in vitro and in vivo validation based on their connectivity score in LINCS analysis effect on TBI-related gene networks and availability in the markets. LINCS connectivity score indicates the magnitude of alterations induced by the compound on post-TBI transcriptomics.

Desmethylclomipramine, an active metabolite of antidepressant clomipramine antidepressant, up-regulates *Nfe2l2*, which promotes anti-inflammatory, antioxidant and neuroprotective proteins [25,26,27]. Ionomycin also upregulated *Nfe2l2* [28]. Trimipramine, a tricyclic antidepressant, modulates *Tp73***,** a transcription factor regulating apoptotic functions [29,30,31]. Sirolimus improves functional recovery and attenuates epileptogenesis after TBI [9,32]. Moreover, desmethylclomipramine, ionomycin and sirolimus have a high LINCS connectivity score and are available on the market.

### 2.1. In Silico Screening of Compounds that Modify TBI-Induced Gene Expression at Both Acute and Chronic Time Points

To predict the therapeutic time window of in vivo treatment, we assessed whether the compounds modifying TBI signature (TBI-sig) at 3 months post-TBI would also modify the TBI-sig at the earlier 32-h time-point. As summarized in Table 1, desmethylclomipramine and ionomycin had a positive concordance value with both the acute and chronic TBI-sig. Trimipramine had a positive concordance value with the acute but not with the chronic, TBI-sig. Sirolimus showed no concordance value with the acute TBI-sig and a negative concordance with the chronic TBI-sig.

### 2.2. Desmethylclomipramine, Ionomycin, Trimipramine and Sirolimus Inhibit TNFα and Nitrite Production in Cortical Neuron-BV2 Microglia Co-Culture

On the basis of the concordance value and its positivity/negativity alone, it is difficult to predict whether a compound will have favourable or unfavourable effects on post-TBI recovery. Therefore, we next investigated whether the four candidate treatments revealed by the iLINCS analysis exhibited anti-inflammatory (reduction in tumour necrosis factor alpha (TNFα) production), anti-oxidant [reduction in nitrite (NO_2_^−^) levels], and/or neuroprotective (neuronal cell survival) properties in in vitro neuronal-microglial co-cultures.

#### 2.2.1. TNFα Secretion

**Figure 2:**
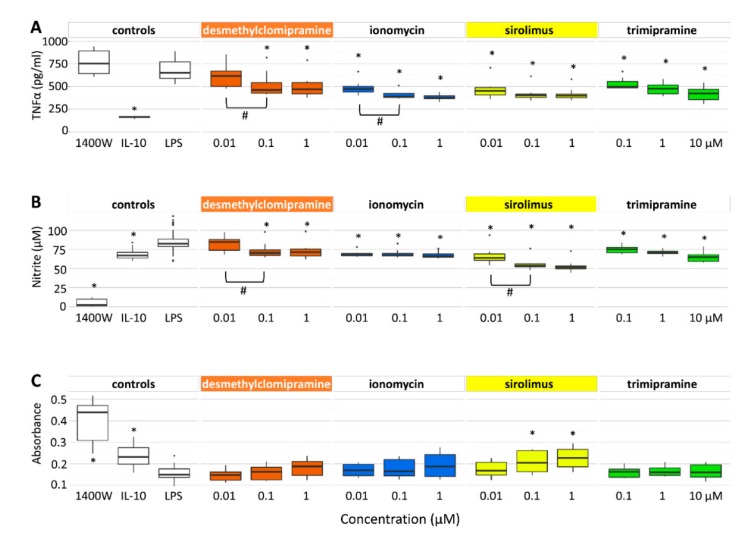
In vitro validation of anti-inflammatory, anti-oxidant and neuroprotective effects of test compounds. (**A**) Treatment effect on neuroinflammation was assessed by measuring TNFα levels in the culture medium as a monitoring biomarker. All test compounds reduced TNFα and the effect was dose-dependent in the cases of desmethylclomipramine and ionomycin. (**B**) The treatment effect on nitric oxide –mediated neurotoxicity was assessed by measuring nitrite levels in the culture medium as a monitoring biomarker. All test compounds also reduced nitrite levels and the effect was dose-dependent in the cases of desmethylclomipramine and sirolimus. (**C**) The treatment effect on neuronal viability was assessed by measuring microtubule-associated protein 2-originated signals in the culture medium as a monitoring biomarker. Only sirolimus improved neuronal viability. Experiments were performed twice, each including quadruplicates of each test compound and concentration. Statistical significance: * *p* < 0.05 (as compared with non-treated control (LPS), Mann-Whitney’s *u*-test) and # *p* < 0.05 dose comparison (Mann-Whitney’s *u*-test). Abbreviations: lipopolysaccharide, (LPS); IL, interleukin; *N*-(3-(aminomethyl)benzyl)acetamidine, 1400 W (inducible nitric oxide synthase inhibitor).

The Mann-Whitney *U*-test revealed that a 48-h exposure of cell cultures to the treatments affected TNFα concentrations in the cell culture media. The TNFα concentration was decreased by desmethylclomipramine (1 µM: *p* = 4.45 × 10^−5^ and 0.1 µM: *p* = 8.25 × 10^−4^), ionomycin (1 µm: *p* = 2.71 × 10^−6^, 0.1 µM: *p* = 2.72 × 10^−6^ and 0.01 µM: *p* = 5.11 × 10−^4^), trimipramine (10 µM: *p* = 5.44 × 10^−6^, 1 µM: *p* = 5.17 × 10^−6^ and 0.1 µM: *p* = 4.60 × 10^−3^) and sirolimus (1 µM: *p* = 3.26 × 10^−5^, 0.1 µM: *p* = 5.17 × 10^−5^ and 0.01 µM: *p* = 5.11 × 10−^4^) compared with untreated controls (Figure 2A, Table S1). The effects of desmethylclomipramine and ionomycin were dose-dependent (0.1 µM being more effective than 0.01 µM in both, *p* < 0.05).

#### 2.2.2. Nitrite Levels

The Mann-Whitney *U*-test indicated that 48-h exposure of cell cultures to the treatments affected the nitrite (NO_2_^−^) concentrations in the cell culture media. The nitrite concentration was decreased by desmethylclomipramine (1 µM: *p* = 5.95 × 10^−4^ and 0.1 µM: *p* = 2.64 × 10^−3^), ionomycin (1 µM: *p* = 6.76×10^−5^, 0.1 µM: *p* = 1.60 × 10−^3^ and 0.01 µM: *p* = 5.12 × 10^−4^), trimipramine (10 µM: *p* = 4.70 × 10^−5^, 1 µM: *p* = 7.63 × 10^−4^ and 0.1 µM: *p* = 2.16 × 10^−2^) and sirolimus (1 µM: *p* = 1.59 × 10^−7^, 0.1 µM: *p* = 2.39 × 10^−7^ and 0.01 µM: *p* = 1.80 × 10^−3^ ) compared with untreated controls (Figure 2B, Table S1). The effects of desmethylclomipramine and sirolimus were dose-dependent (0.1 µM being more effective than 0.01 µM in both, *p* < 0.05).

#### 2.2.3. Neuronal Viability

The Mann-Whitney *U*-test revealed increased neuronal viability (*p* < 0.05) following treatment with sirolimus (1 µM: *p* = 4.46 × 10^−4^, 0.1 µM: *p* = 1.12 × 10^−2^) but desmethylclomipramine, ionomycin or trimipramine, compared with untreated controls (Figure 2C, Table S1).

These data showed that sirolimus had a favourable effect on all three outcome measures. Desmethylclomipramine, ionomycin and trimipramine reduced neuroinflammation and nitrite production but did not modify neuronal survival.

### 2.3. Target Gene Expression After TBI in Vivo and Effect of Candidate Compounds on Target Gene Expression In Vitro

#### 2.3.1. Target Gene Expression after TBI In Vivo

First, we verified that the TBI-sig genes predicted to be targeted by candidate treatments in silico were regulated by TBI in vivo with a focus on neuroinflammation (cytokines) and neurotoxicity (nitric oxide [NO] production). As summarized in Table 2, lateral fluid-percussion injury (FPI) induced an upregulation of the Nrf2 target genes *Hmox1* and *Nqo1* in the perilesional cortex at 32 h and at 3 months post-TBI. *Gclm* expression was upregulated only at 32 h and *Nfe2l2* was upregulated only at 3 months post-TBI. Nitric oxide synthase gene *Nos2* was downregulated at 32 h and upregulated at 3 months post-TBI.

To confirm the co-occurrence of neurotoxicity and neuroinflammation in vivo, we investigated the expression levels of the inflammatory genes in the same samples. Of 12 cytokines, 10 had altered gene expression in the perilesional cortex at 32 h post-TBI, including upregulation of *Il1b* and *ll6* and downregulation of *Il1a*, *Il2*, *Il4*, *Il5*, *Il10*, *Il12a*, *Il-13*, *Csf2* and *Ifng*. Expression of and *Tnf* was not altered. Three months post-TBI, none of the 12 cytokines showed altered gene expression in the perilesional cortex (Table 2). These data indicated that neurotoxicity and cytokine responses co-occurred during the acute phase. Note that the plasma levels of the corresponding proteins were investigated in the acute post-TBI phase in vivo as predictive biomarkers for a therapeutic response (see below).

Effect of candidate treatments on target gene expression in BV2 microglia cultures under non-inflammatory and inflammatory conditions. We then assessed whether the candidate treatments modulated target gene expression under inflammatory (cf. acute TBI tissue) and non-inflammatory (cf. chronic TBI tissue) conditions in vitro. We were particularly interested in the expression of the *Nfe2l2* transcription factor and its activation, which our previous in silico analysis predicted to be regulated by desmethylclomipramine and ionomycin [23] and which we validated in vivo (see Section 4.3). Moreover, increased expression or availability of Nrf2 is neuroprotective and improves post-injury recovery [33,34,35].

#### 2.3.2. Inflammatory Condition

Desmethylclomipramine upregulated *Nfe2l2* gene expression (*p* = 1.87 × 10^−2^) in BV2 microglial cultures under lipopolysaccharide/interferon gamma (LPS/IFNγ)-induced inflammation (Table 3). It did not change the expression of the Nrf2 target genes, *Gclm* (*p* = 7.70 × 10^−1^), *Nqo1* (*p* = 7.85 × 10^−1^) and *Hmox1* (*p* = 6.64 × 10^−1^) (Table 3).

Ionomycin had no effect on *Nfe2l2* (*p* = 7.87 × 10^−1^) or its target genes *Gclm* (*p* = 1.04 × 10^−1^ ) and *Hmox1* (*p* = 1.54 × 10^−1^) under inflammatory conditions but upregulated *Nqo1* (*p* = 8.72 × 10^−3^) (Table 3).

#### 2.3.3. Non-inflammatory Condition

Desmethylclomipramine upregulated *Nfe2l2* gene expression (*p* = 3.83 × 10^−2^) in BV2 microglial cultures under non-inflammatory conditions (Table 3). It did not change the expression of the *Nfe2l2* target genes *Gclm* (*p* = 7.83 × 10^−1^), *Hmox1* (*p* = 3.99 × 10^−1^) and *Nqo1* (*p* = 1.09 × 10^−1^) (Table 3).

Ionomycin did not change expression of *Nfe2l2* (*p* = 1.65 × 10^−1^), *Gclm* (*p* = 4.52 × 10^−1^), *Hmox1* (*p* = 6.58 × 10^−2^) or *Nqo1* (*p* = 8.88 × 10^−2^) (Table 3) under non-inflammatory conditions.

To summarize, desmethylclomipramine was selected for further testing as it exhibited both anti-oxidant and anti-inflammatory effects and regulated target gene expression in vitro. In particular, desmethylclomipramine also upregulated *Nfe2l2* gene expression under inflammatory conditions. Moreover, clomipramine, which is rapidly metabolized to desmethylclomipramine, passes through the blood-brain barrier and is water soluble [36,37], allowing it to be chronically administered via osmotic mini-pumps to achieve stable therapeutic tissue levels.

### 2.4. Scoring of Candidate Compounds to Proceed to In Vivo Proof-of-Concept Validation

The properties of the four candidate compounds were scored to make a Go/No-go decision to proceed to an in vivo proof-of-concept validation trial. The compound properties included in the scoring were (a) in silico-predicted therapeutic time window, (b) pharmacokinetics (blood brain barrier penetration and water solubility), (c) effect on monitoring biomarkers (anti-inflammatory, nitric oxide-mediated neurotoxicity and neuroprotective) in the co-culture and (d) target engagement under inflammatory and non-inflammatory conditions. Using this scoring system, desmethylclomipramine scored 1.775 points and ionomycin scored 0.2625 points (Table 4).

### 2.5. Clomipramine Treatment in the Acute Post-TBI Phase Had a Negative Effect on Somatomotor Recovery

#### 2.5.1. Acute Mortality, Impact Pressure, Post-impact Apnoea and Acute Post-impact Seizure-like Behaviour

The acute (<48 h) post-TBI mortality was 13.3%. The impact pressure did not differ between the Vehicle-TBI (3.29 ± 0.13 atm, range 3.08–3.48 atm) and Clomi-TBI (3.33 ± 0.08 atm, range 3.21–3.50 atm) groups (*p* = 6.78 × 10^−1^). The duration of post-impact apnoea did not differ between the groups [22.7 ± 10.3 s (range 15–45 s) versus 32.7 ± 9.6 s (range 20–45 s), *p* = 2.08 × 10^−1^, respectively]. Also, duration of acute post-impact seizure-like behaviour was comparable [11 *vs.* 15.6 s, (range 11.0–40.0 s) versus 8.2 ± 14.2 (range 0.0–35.0 s), *p* = 5.40 × 10^−1^].

#### 2.5.2. Pathological Outcome

Visual analysis of thionin-stained sections revealed that the location of the cortical lesion was comparable between the Vehicle-TBI and Clomi-TBI groups. Moreover, in both groups the progression of lesion extent was largely variable (Figure S1).

#### 2.5.3. Composite Neuroscore

**Figure 3:**
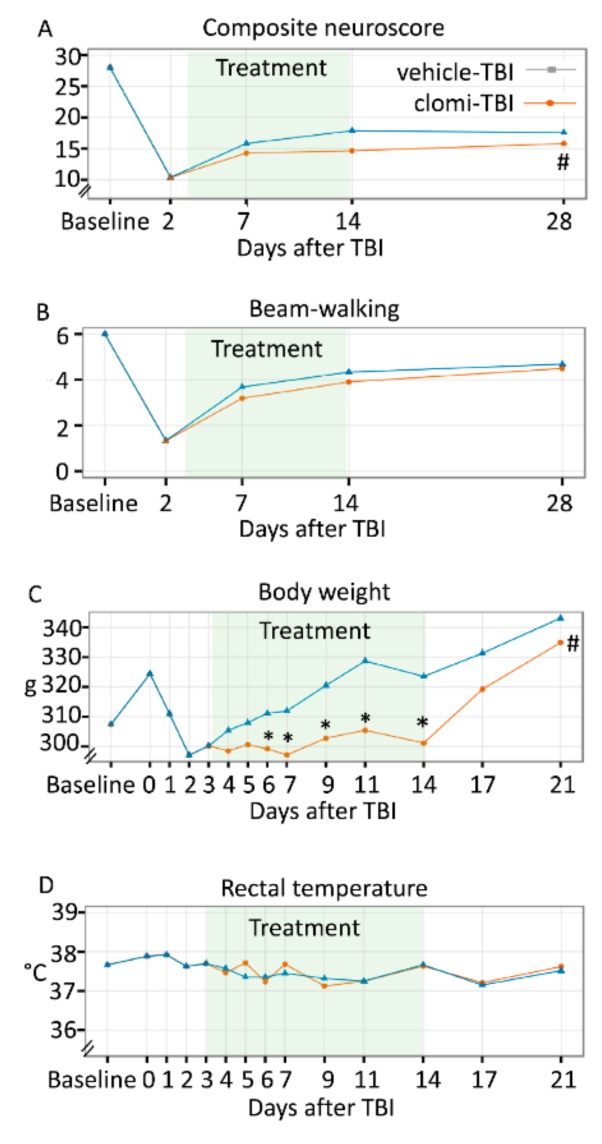
Effect of test compounds on in vivo functional outcomes. Effects of vehicle (*n* = 15) and clomipramine (Clomi, *n* = 11) treatments on composite neuroscore, beam-walking, body weight and rectal temperature in rats with traumatic brain injury (TBI) were analysed using coefficient estimates of the linear mixed effects model. The treatment period (3–14 days) is indicated with a light green area in each panel. (**A**) Clomipramine administration impaired performance in the composite neuroscore test compared with vehicle treatment. (**B**) Performance in the beam-walking test was comparable between the Vehicle-TBI and Clomi-TBI groups. (**C**) Clomipramine delayed the post-TBI weight gain during the 3–14-days treatment period. After clomipramine discontinuation, rats stated to gain weight and by 21 days post-TBI, their weight did not differ from that in the vehicle group. (**D**) Rectal temperature was comparable in the Vehicle-TBI and Clomi-TBI groups throughout the entire study period. Statistical significance from: ^#^ *p* < 0.05 (significant main effects model); * *p* < 0.05 (significant estimates in model).

The linear mixed effect model indicated a significant main effect of treatment on the composite neuroscore (*p* = 5.40 × 10^−3^). Compared with the Vehicle-TBI group, the estimated effect of Clomi-TBI treatment on the neuroscore was −2.2 points (*p* = 5.40 × 10^−3^). Thus, performance in the neuroscore test was impaired in the Clomi-TBI group between 7–28 days post-TBI (Figure 3A).

#### 2.5.4. Beam-walking

The linear mixed effect model did not indicate any significant main effect on beam-walking (*p* = 4.27 × 10^−1^). Thus, clomipramine treatment did not affect performance in the beam-walking test after TBI (Figure 3B).

### 2.6. Clomipramine Treatment Did Not Affect Spatial Learning or Spatial Memory

#### Morris Water-maze

The Cox proportional hazards model did not reveal any treatment effect on the latencies in the acquisition phase (*p* = 5.35 × 10^−1^), indicating that clomipramine treatment did not affect spatial learning (Table S2). The Kruskal-Wallis test detected no difference between the treatment groups in the probe trial latency at 3 days (*p* = 5.71 × 10^−1^) or 5 days (*p* = 2.50 × 10^−1^), indicating that clomipramine did not affect spatial memory. Swimming speed did not differ between the Vehicle-TBI and Clomi-TBI groups (*p* > 0.05).

### 2.7. Clomipramine Treatment Reduced Weight Gain

#### 2.7.1. Body Weight

The linear mixed effects model indicated a significant interaction between body weight and duration of clomipramine treatment (*p* = 3.12 × 10^−6^) compared with the Vehicle-TBI group. Model estimate of clomipramine treatment on body weight was −5.1 g at 6 days (*p* = 2.77 × 10^−3^), −7.9 g at 7 days (*p* = 2.10 × 10^−4^), −10.8 g at 9 days (*p* = 3.00 × 10^−5^), −16.5 g at 11 days (*p* = 1.50 × 10^−7^) and −15.5 g at 14 days after TBI (*p* = 4.82 × 10^−6^), indicating reduced post-TBI weight gain (Figure 3C). During the post-treatment period, model estimates of the clomipramine effect were non-significant (17 days; *p* = 9.74 × 10^−2^, 21 days; *p* = 1.40 × 10^−1^), indicating that the body weight of Clomi-TBI animals caught up to that of the Vehicle-TBI animals.

#### 2.7.2. Rectal Temperature

The linear mixed effects model revealed no treatment effect on rectal temperature (*p* = 2.73 × 10^−1^), indicating that clomipramine treatment did not affect rectal temperature compared with vehicle treatment (Figure 3D).

#### 2.7.3. General Health

Pearson’s Chi-squared test detected no difference in the number of health problems (*p* > 0.05) between the Vehicle-TBI and Clomi-TBI groups during the treatment (3–14 days) or post-treatment (15–28 days) periods.

### 2.8. Clomipramine Treatment Delayed the Reduction of the TBI-Induced Plasma Cytokine Response

#### 2.8.1. Plasma Cytokine Response to Injury and Clomipramine

The linear mixed effects model revealed no overall treatment effect on any of the plasma cytokines analysed (GM-CSF, IFNy, IL-1α, IL-1β, IL-2, IL-4, IL-5, IL-6, IL-10, IL-12p70, IL-13 or TNFα), indicating that clomipramine treatment did not modify post-TBI cytokine plasma concentrations. A model analysing the association between the cytokine concentrations and post-TBI day indicated a significant time-dependent variation (*p* < 0.05) in IFNy, IL-1α, IL-1β, IL-2, IL-4, IL-5, IL-6, IL-10, IL-12p70, IL-13 and TNFα concentrations. Model estimates indicated that the cytokine concentrations were elevated at 7 days and 14 days post-TBI compared with those at 28 days post-TBI in both the Vehicle-TBI and Clomi-TBI groups (Table S3).

Interestingly, the more severe the impairment before drug initiation, that is, the lower the neuroscore at 2 days, the higher the cytokine levels at 7 days in the Vehicle-TBI group. No association was found between the neuroscore at 2 days and the cytokine levels at 7 days in the Clomi-TBI group. The effect was detected for all plasma cytokines measured. Moreover, the better the recovery, that is, the better the performance in the neuroscore and/or beam-walking tests at 7 d, the higher the cytokine levels at 14 and 28 days in the Clomi-TBI group (Table S4). Even though we found some correlations between the functional performance and plasma cytokine levels, there were no group differences in individual cytokines. Therefore, we next compared the plasma cytokine patterns between the treatment groups using machine learning.

#### 2.8.2. Machine Learning Separated Clomipramine- and Saline-treated Animals at 28 days post-TBI

**Figure 4:**
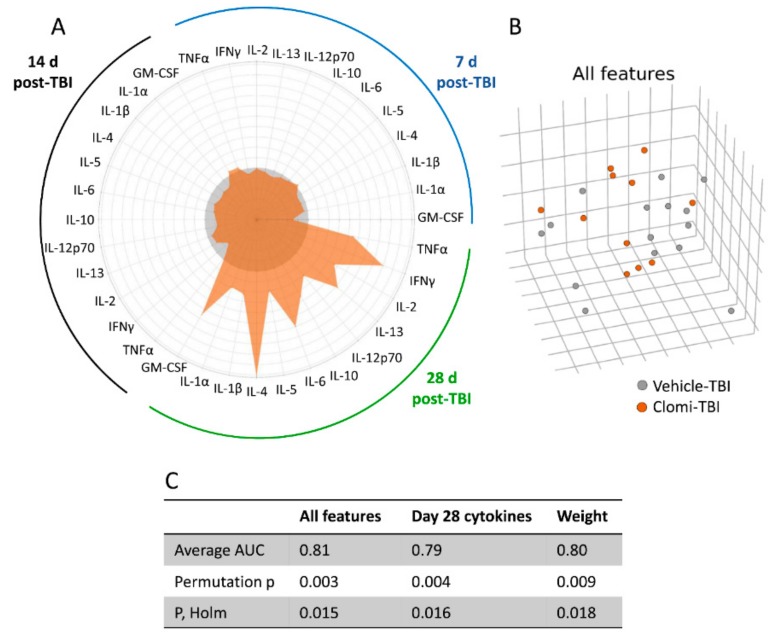
Effect of clomipramine on plasma cytokines and machine learning analysis. (**A**) A radar plot showing the levels of plasma cytokines in clomipramine (Clomi)-treated (orange, *n* = 11) and vehicle-treated (grey, *n* = 15) rats with lateral fluid-percussion-induced traumatic brain injury (TBI) at 7 days, 14 days, 28 days post-TBI. Mean concentrations were obtained from the linear mixed effect model (Table S3). Note that machine learning differentiated Vehicle-TBI and Clomi-TBI rats by all tested cytokines at 28 days post-TBI. (**B**) 3D dot-plots showing the differentiation of Vehicle-TBI and Clomi-TBI rats in machine learning analysis by all features [area under curve (AUC) 0.81]. (**C**) Summary of machine leaning analysis of Vehicle-TBI and Clomi-TBI groups. Average AUC score from 10 cross-validation. Machine learning differentiated Vehicle-TBI rats (grey) and Clomi-TBI (orange) (AUC 0.81).

Further analysis applying machine learning (ML), using gradient boosted trees as a classifier, differentiated the Vehicle-TBI and Clomi-TBI groups by all cytokines at 28 days post-TBI (AUC 0.79) (Figure 4A,C). The mean concentration of all cytokines was higher in the Clomi-TBI group than in the Vehicle-TBI group (Figure 4A, Table S3), indicating that clomipramine delayed the decline of plasma cytokine levels. As found above using a linear mixed model, ML did not separate the Vehicle-TBI and Clomi-TBI groups based on plasma cytokine levels at 7 days (AUC 0.39) or 14 days (AUC 0.62) post-TBI.

ML analysis separated the Vehicle-TBI and Clomi-TBI groups by all features (AUC 0.81) (Figure 4B), indicating a clomipramine treatment effect on the outcome measures. As found above using the linear mixed model, ML analysis separated the Vehicle-TBI and Clomi-TBI groups by body weight (AUC 0.80) (Figure 4C).

## 3. Discussion

Our previous in silico analysis predicted desmethylclomipramine, ionomycin, trimipramine and sirolimus (rapamycin) as candidate treatments for modulation of post-TBI gene expression based on their interaction with anti-inflammatory and anti-oxidant gene networks [22,23]. However, the net effect of the compounds on post-injury functional outcome in vivo is difficult to predict based on in silico analysis only. Also, anticipation of adverse events based on in silico data is a challenge. Adverse events can, however, negligible effect on the overall outcome both in animal models and humans. Therefore, our objective was to develop a pipeline for validation of in silico predictions in vitro and assessment of their favourable as well as unfavourable disease-modifying effects on post-TBI outcome in an in vivo proof-of-concept preclinical trial.

### 3.1. In Silico Analysis Predicted a Wide Therapeutic Time-Window for Desmethylclomipramine and Ionomycin

We first explored the time window of transcriptomics regulation for each of the four candidate compounds. iLINCS analysis revealed that desmethylclomipramine modulated TBI-induced gene expression at both 32 h and 3 months post-TBI, suggesting a wide therapeutic time-window. There are no prior predictions on the functional effects of transcriptomics regulation by desmethylclomipramine. Our literature review, however, suggested that transcriptomics regulation by desmethylclomipramine could be beneficial, as the effect of its parent drug clomipramine on gene expression in MCF7 cultures was comparable to that of compound F05, which promoted axonal regeneration in a rat optic nerve crush model [38,39].

Of the other three compounds, ionomycin also modulated TBI-induced gene expression at both the 32-h and 3-month time-points, trimipramine had an acute effect and sirolimus demonstrated an effect at only the chronic time-point. We found no prior in silico studies on target gene networks for ionomycin or trimipramine. Also, in silico analyses of sirolimus-induced transcriptomics changes in neuronal tissues are sparse. One study using Connectivity Map (CMap) analysis reported a connection between the transcriptomic profile of sirolimus and “pro-oligodendrogenic,” predicting an effect on gene networks promoting oligodendrogenesis [40].

In conclusion, in silico analysis suggested that desmethylclomipramine and ionomycin would modulate TBI-induced transcriptomics changes at both acute and chronic post-TBI time periods. The little information available on the functional consequences of the transcriptomics modulation suggested a favourable outcome.

### 3.2. In Vitro Analysis Revealed that Desmethylclomipramine, Ionomycin, Trimipramine and Sirolimus Reduce Neuroinflammation and Nitric Oxide—Mediated Neurotoxicity

Based on in silico analysis only, it is difficult to predict whether the positive or negative concordance score revealed by the iLINCS analysis is associated with positive or negative effects on post-TBI secondary pathologies. Therefore, we performed an in vitro analysis to assess the treatment effects on neuroinflammation and neurodegeneration, which are the major components of post-TBI secondary tissue damage [4]. As monitoring biomarkers, we used TNFα for neuroinflammation, nitrite for nitric oxide –mediated neurotoxicity and MAP2 for neurodegeneration in neuron-BV2 microglia co-cultures stressed by LPS/IFNγ-induced inflammation [41].

Desmethylclomipramine reduced TNFα levels by 12%–26%, an effect that was dose-dependent at 0.01–0.1 μM concentrations. It also reduced NO_2_^−^ levels by 27% at 0.1–1 μM concentrations. Neuronal viability, however, was not affected. In a PubMed search, we were unable to identify any prior studies investigating the biologic effects of desmethylclomipramine in neuronal cell culture models. Clomipramine, the parent drug of desmethylclomipramine, however, reportedly decreased TNFα and nitric oxide production at 10–20 µM concentrations in LPS-activated mouse BV2-microglia culture [42]. Clomipramine also showed a dose-dependent neuroprotective effect on FeSO_4_-mediated neurotoxicity in human primary neuronal cultures at concentrations from 0.1–10 µM [43]. It also improved neuronal viability in LPS-activated mouse BV2-microglia cultures at concentrations of 10–20 µM [42]. Taken together, at concentrations comparable to those of therapeutic antidepressant plasma, both clomipramine (rat 0.1–0.5 µM; human 0.3–1.1 µM) and desmethylclomipramine (rat 0.16–1.3 µM; human 0.2–1.8 µM) reduced neuroinflammation, nitrite formation and neurodegeneration in cell culture models [37,44,45,46,47].

Ionomycin dose-dependently reduced TNFα levels by 29%–44% at concentrations of 0.01–1 µM. It also reduced NO_2_^−^ levels by 17% at concentrations of 0.01–1 µM. Neuronal viability, however, was not affected in vitro in neuron BV2-microglia co-culture. Hutter-Paier et al. (2000) [48] reported reduced neuronal viability of primary cortical neurons isolated from chick embryos at ionomycin concentrations comparable to those used in our study, suggesting a cell-type specificity of the effect. Ionomycin has not been administered in vivo and thus there is no information on its blood-brain barrier permeability or therapeutic concentrations.

Trimipramine reduced TNFα production by 30% and NO_2_^−^ by 15% at concentrations of 0.1–10 µM, which are comparable to its therapeutic antidepressant plasma concentration in humans (0.2–0.6 µM; Regenthal et al., 1999) [49]. Like desmethylclomipramine and ionomycin, trimipramine did not affect neuronal viability in the co-culture model. Trimipramine at a concentration of 10 µM was found to reduce iron neurotoxicity in human primary neuronal cultures [43].

Sirolimus reduced TNFα levels by 39% at concentrations of 0.01–1 µM. It also reduced NO_2_^−^ levels by 10%–22% and the effect was dose-dependent at concentrations of 0.01–0.1 μM. Interestingly, sirolimus counteracted the increased nitric oxide production in rat primary microglia culture at concentrations of 0.5–5 nM when inflammation was activated by a mixture of pro-inflammatory cytokines (IFNγ, TNFα and IL-1β) [50]. No effect was detected at the concentrations used in the present study (0.1–1.0 nM) [50]. Unlike other compounds tested, sirolimus increased neuronal viability by 35%–47% at concentrations of 0.1–1 µM. Consistent with the anti-inflammatory, anti-oxidant and neuroprotective effects detected in vitro, intraperitoneal administration of 0.5 or 1 mg/kg sirolimus at 4 h after weight-drop–induced TBI in mice was neuroprotective and reduced the inflammatory response and microglia activation when assessed at 3 days post-injury [9]. Interestingly, the immunosuppressive plasma concentrations of sirolimus (0.01–0.2 µM) used to prevent rejection of organ transplants in pre-clinical and clinical studies correspond to those used in the present and previous in vitro studies [51].

Taken together, all four compounds exhibited favourable in vitro effects on neuroinflammation and nitrite formation. In addition, sirolimus was neuroprotective.

### 3.3. Target Expression and Target Engagement

Next, we analysed the in vivo cortical post-TBI transcriptomics data at 32 h and 3 months to pinpoint gene expression changes that could be used as indicators of target gene engagement by test treatments. Positive concordance scores in our previous in silico and gene network analyses predicted that both desmethylclomipramine and ionomycin (but not trimipramine or sirolimus) would upregulate the expression of *Nfe2l2* [23], suggesting a mechanistic role for the Nrf2 transcription factor in their treatment effects. The present transcriptomics analysis showed a 51% chronic upregulation of *Nfe2l2* in the perilesional cortex after TBI. Importantly, Nrf2 promotes the expression of genes encoding anti-oxidant proteins [52], particularly *Hmox1* and *Nqo1,* which were acutely upregulated after TBI by 726% and 134% and chronically upregulated by 227% and 156%, respectively. Further, it is well known that activation or increased levels of Nrf2 favourably modify post-injury tissue pathology and functional recovery in brain injury models, including chemoconvulsant-induced status epilepticus [33,34,53], TBI [54,55] and stroke [56,57,58]. Therefore, we selected *Nfe2l2* and Nrf2 target genes as indicators of target engagement for desmethylclomipramine and ionomycin treatments in vitro.

*In vitro* analysis of target engagement indicated that desmethylclomipramine indeed upregulated *Nfe2l2* gene expression by 115%–121% already after 16-h incubation in both inflammatory and non-inflammatory conditions. Although desmethylclomipramine did not regulate the expression of Nrf2 target genes, we would expect the desmethylclomipramine-induced augmented *Nfe2l2* expression to have favourable in vivo effects.

We found no major ionomycin effect of target gene expression in a 16-h incubation paradigm.

Taken together, compared with ionomycin, desmethylclomipramine showed target engagement under both inflammatory and non-inflammatory conditions.

### 3.4. Go/No-Go Analysis for Selection of a Compound for In Vivo Testing

Selection of the compound for the in vivo phase was based on weighted scoring, taking into account: (a) in silico-predicted wide therapeutic time window; and (b) pharmacokinetic properties of the compound, particularly the blood-brain barrier penetration and water solubility of the compound, enabling subcutaneous minipump administration to achieve stable plasma/brain concentrations; (c) anti-inflammatory, anti-oxidant and neuroprotective properties; and (d) target gene network engagement. Higher the score, better the expected in vivo performance. Of the four test compounds, desmethylclomipramine had the highest score. Depression is a common co-morbidity after TBI and is treated with antidepressants, including tricyclic antidepressants such as clomipramine [59,60]. To date, however, almost no data is available on the disease-modifying effects of post-TBI antidepressant treatments.

Desmethylclomipramine is an active metabolite of the antidepressant clomipramine in rodents and primates and crosses the blood-brain barrier [37,45,46]. It is water soluble and its therapeutic plasma and brain concentrations are established [36,37,46]. Data on its adverse events are also reported [61]. Importantly, these adverse events do not include effects on blood pressure or intracerebral pressure, which would be detrimental considering future clinical use. Our in silico analysis predicted a wide therapeutic window for desmethylclomipramine. In vitro analysis indicated a reduction in neuroinflammation and nitric oxide –mediated neurotoxicity. Upregulation of *Nfe2l2* in inflammatory and non-inflammatory conditions indicated target gene network engagement. Importantly, *Nfe2l2* encodes the Nrf2 transcription factor and its activation or upregulation has favourable effects on different types of brain injuries; therefore, it could favourably modify post-TBI outcome [54,55,57,58].

Ionomycin was excluded from further in vivo validation as it did not show regulation of *Nfe2l2* in the target engagement analysis. Some previous data, however, suggested that it reduced neuronal survival in some cell culture models [48] and its pharmacokinetics and blood-brain barrier permeability are unknown. It is soluble in chloroform and dimethyl sulfoxide (DMSO) (10 mg/mL), which limits its administration via osmotic minipump. Trimipramine and sirolimus were withdrawn from the in vivo phase due to the narrow therapeutic time window for their effect on post-TBI gene expression predicted by in silico analysis.

### 3.5. In Vivo Assessment Failed to Demonstrate Favorable Disease-Modifying Effects of Clomipramine on TBI Functional Outcome and Plasma Cytokines

To assess the post-TBI therapeutic properties of clomipramine, we induced lateral FPI in rats and initiated clomipramine treatment via subcutaneous minipumps to ensure a stable therapeutic concentration. As expected, clomipramine slowed down the post-injury weight gain of the TBI animals, which is a well-known adverse event associated with clomipramine use [62,63,64]. Contrary to our expectations based on the favourable findings in the in silico and in vitro analyses, clomipramine impaired somatomotor recovery after TBI in the neuroscore test—An effect that persisted even after treatment discontinuation. The poor performance could relate to dizziness and sedation, which are well-known side effects of clomipramine in humans [61]. Recently, we found that rats with lateral FPI have non-convulsive status epilepticus lasting approximately 3 days [65]. Thus, some of our animals could still have had epileptiform activity when the clomipramine treatment was initiated. Because tricyclic antidepressants, including clomipramine, are known to reduce the seizure threshold in animal models and humans [66,67,68], worsening of post-TBI epileptiform activity and its influence on the evolution of secondary pathologies must be taken into account in future studies. The effect appeared to be test-dependent, however, as no impairment was observed in the beam-walking test performed on the same day as the neuroscore test. Compared with vehicle treatment, clomipramine treatment exhibited no beneficial or harmful effects on spatial learning in rats with TBI when tested at approximately 2 weeks after treatment discontinuation.

Previous studies demonstrated a robust increase in brain cytokine levels after lateral FPI and other types of experimental brain injuries and reported an association with favourable or unfavourable injury outcomes [69]. Moreover, the circulating levels of cytokines are elevated post-TBI in both rat models and humans [70,71,72,73,74,75] and may be promising predictive biomarkers of a therapeutic response [76,77,78,79]. As clomipramine has been reported to reduce brain cytokine levels after brain injury, we hypothesized that normalization of elevated plasma TNFα and/or other cytokine levels could serve as a predictive biomarker for clomipramine treatment. In particular, our data showed that desmethylclomipramine reduced TNFα levels in an in vitro assay in a dose-dependent manner, suggesting that clomipramine, which is metabolized to desmethylclomipramine in vivo, could be anti-inflammatory after TBI [37]. Unexpectedly, however, ML analysis indicated that the overall pattern of plasma cytokines remained elevated in the Clomi-TBI group even at 28 days after TBI compared with the Vehicle-TBI group, that is, for up to 2 weeks after clomipramine discontinuation. To our knowledge, there are no data on the effect of clomipramine on plasma cytokine levels. It remains to be explored, whether clomipramine directly affects the plasma cytokines or whether a prolonged elevation in plasma cytokine levels reports on prolonged TBI-induced tissue inflammation.

Several lessons were learned after we found that despite favourable in silico and in vitro data, clomipramine administered in the subacute post-TBI phase did not improve functional outcome post-TBI. Rather, animals exhibited weight loss, poor performance in the composite neuroscore test and prolonged elevation of plasma cytokines. First, the in vitro methodological test platform can be expanded, for example, by increasing the types of cell culture models. Second, also the number of in vivo tests can be increased, particularly towards a more extensive assessment of adverse events, which can mask the favourable treatment effects and even result in false negative findings. Third, scoring and weighting the individual scores need to be revised after adding new variables to the equation. Fourth, time window of treatment administration needs to be matched with the spatio-temporal expression of treatment targets. Fifth, criteria should be set for monitoring and predictive biomarkers to decide when the treatment effect is favourable. Moreover, monitoring biomarkers for adverse events should be included. Sixth, the pipeline can be expanded to also include the other animal models, strains and gender. Overall, the present study emphasizes the importance of in vivo assessment of in silico identified compounds, including the analysis of adverse events.

## 4. Materials and Methods

The screening pipeline is outlined in Figure 1.

### 4.1. In Silico Analysis of Transcriptomic Modifying Compounds

#### 4.1.1. The Effect of Test Compounds on Acute and Chronic TBI Transcriptomic

*iLINCS analysis of acute and chronic transcriptomic signatures.* To expand our previous analysis [22,23] and assess whether desmethylclomipramine, ionomycin, sirolimus and trimipramine would modulate post-TBI transcriptomics changes in the perilesional cortex, acute and chronic TBI signature (TBI-sig) (see below) were submitted to the analysis service of the LINCS data portal [80] and compared with LINCS chemical perturbagen signatures. As outcomes, we expected the four compounds to show high +/− concordance scores at both time-points.

*Generation of acute (32 h) TBI-sig for the perilesional cortex*. The “acute cortical TBI-sig” was generated using 32-h post-TBI microarray data [81]. Rat phalanx gene IDs were translated to NCBI human IDs with BioMart. The differentially expressed genes (fold change > 1.5 and Benjamini-Hochberg false discovery rate < 0.05) were then included in the acute TBI-sig.

*Generation of chronic (3 months) TBI-sig for the perilesional cortex*. The “chronic cortical TBI-sig” was generated using 3-month post-TBI RNA-seq data (GEO; series accession number GSE80174), which was mapped with the Spliced Transcripts Alignment to Reference (STAR) aligner (version 2.3.0e_r291) to the rat reference genome (Ensembl Rnor_6.0) [82]. Differentially expressed genes were then identified with the DEseq2 R-package [83] as described in Lipponen et al. (2016) [23]. Rat ensemble gene IDs were translated to human NCBI gene IDs with BioMart (www.enssembl.org/biomart/martview) and differentially expressed genes (fold change >1.5 and Benjamini-Hochberg false discovery rate <0.05) were included in the chronic TBI-sig.

#### 4.1.2. In Silico Identification of Monitoring Biomarkers for in Vitro and in Vivo Analyses.

The 32-h and 3-month perilesional cortical transcriptomics signatures were analysed to identify regulated genes that would report on ongoing post-TBI pathology and indicate target engagement by the treatments in in vitro and in vivo experiments. We focused on genes involved in inflammation and nitric oxide production.

### 4.2. In Vitro Validation of in Silico Discovered Compounds

**Figure 5:**
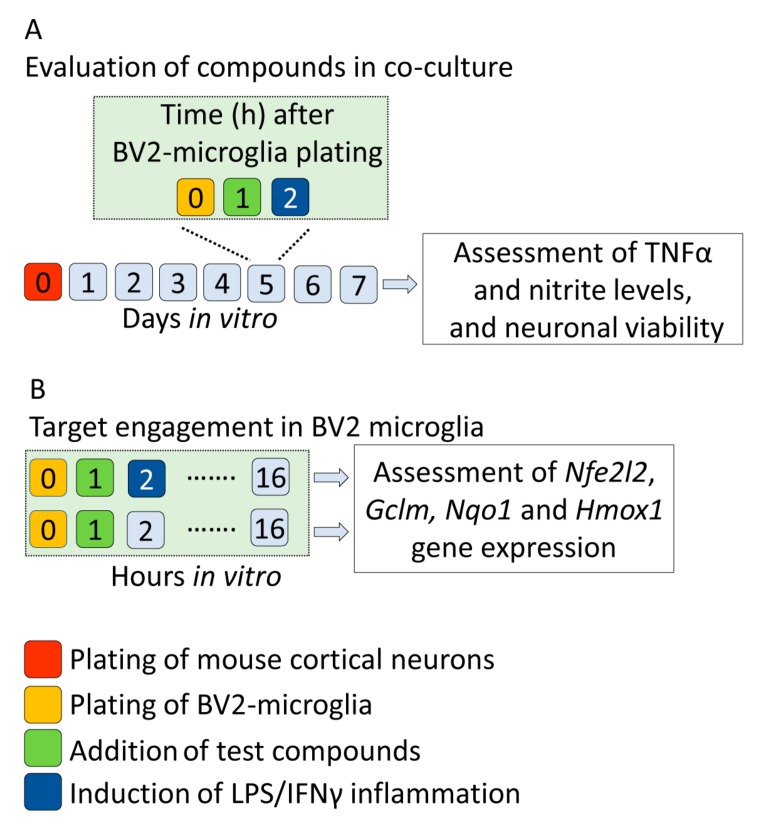
In vitro study design for assessment of anti-inflammatory, anti-oxidant and neuroprotective effects in neuron-BV2 microglia co-culture, as well as target engagement in BV2 microglia. (**A**) Co-culture experiment. Day 0: dissection of brains and plating of mouse cortical neurons; day 5 in vitro: addition of BV2-microglia to prepare co-culture, addition of test compounds to co-cultures and induction of inflammation by lipopolysaccharide/interferon gamma (LPS/IFNγ); day 7 in vitro: collection of culture media for tumour necrosis factor alpha (TNFα) and nitrite (NO_2_^−^) analysis, fixation of co-culture cells to culture plates for assessment of neuronal viability. (**B**) Target engagement. At 1 h after BV2-microglia plating, desmethylclomipramine or ionomycin was added to BV2 cultures. One hour later, inflammation was induced with LPS/IFNγ to one set of cells and another set of cells was left without inflammation. After 16 in vitro, target expression was assessed by quantifying the effects of the treatments on the expression of *Nfe2l2* and Nrf2 target genes (*Gclm*, *Nqo1* and *Hmox1*). Both experiments were performed twice, each including quadruplicates.

Figure 5 summarizes the study design for in vitro experiments.

#### 4.2.1. Preparation of Neuron-BV2 Co-Cultures

Previous evidence from our and other laboratories show that neuron-BV2 co-cultures are useful tool test neuroprotective and anti-inflammatory compounds [41,84,85]. Therefore, co-cultures were prepared from primary mouse cortical neurons and BV2 microglia as described previously [41,84]. Briefly, the heads of mouse embryos were collected on embryonic day 18 (JAXC57BL/6J, Lab Animal Centre of University of Eastern Finland). The cerebral cortex was dissected under a stereomicroscope in Dulbecco’s modified Eagles medium (DMEM; #BE12-614F, Lonza, Basel, Switzerland), supplemented with 10% foetal bovine serum (FBS; #10270-106, Thermo Fisher Scientific, Waltham, MA, USA) and 2 mM l-glutamine (#BE17-605E, Lonza). Cortices were rinsed with HC-buffer (phosphate-buffered saline [PBS], 1 mg/mL bovine serum albumin [BSA], 10 mM glucose) and trypsin-digested for 20 min at 37 °C with 0.125% trypsin-DMEM. Trypsin (#15090-046, Thermo Fisher Scientific) digestion was stopped with an equivalent volume of DMEM containing 10% FBS, 100 U/mL penicillin and 100 μg/mL streptomycin (#DE17-602E, Lonza). The tissue was centrifuged (1600× *g*, 5 min), suspended in plating medium with a pipette and incubated at room temperature for 2 min to obtain a single-cell suspension. The cell suspension was filtered through a 40-μm filter to remove the remaining tissue and then centrifuged (1200× *g*, 5 min) to remove the plating medium. Neurons were plated on poly-d -lysine-coated (#P6407, Sigma-Aldrich, St. Louis, MO, USA) 48-well plates in neurobasal-medium supplemented with B27 (#17504-044, Thermo Fisher Scientific) penicillin, streptomycin and l-glutamine at a density of 2 × 10^5^ cells/well in volume of 500 µL. On day 5 in vitro, 350 µL of old medium was removed and BV2 cells were added to prepare co-cultures, as described previously by Gresa-Arribas et al. [41]. Briefly, BV2 cells were cultured in RPMI-1640-medium (#R0883, Sigma-Aldrich) containing 10% FBS, 2 mM l-glutamine, 100 U/mL penicillin and 100 μg/mL streptomycin. BV2 cells were gently detached with a cell scraper into fresh neurobasal medium and added to primary cortical neuron cultures in a volume of 200 µL at a BV2:neuron ratio of 1:5 and allowed to attach for 1 h.

#### 4.2.2. Treatment of Co-Cultures with Test Compounds

One hour after adding the BV2 microglia to the neuronal culture, the co-cultures were treated with one of four concentrations of the test compounds. *Desmethylclomipramine* (#N1280, Sigma-Aldrich) was dissolved in dimethyl sulfoxide (DMSO; #D2650, Sigma-Aldrich) at a concentration of 10 mM. The stock solution was then diluted with cell culture medium to obtain 1000-µM, 100-µM, 10-µM and 1-µM solutions. *Ionomycin* (#I0634, Sigma-Aldrich) was dissolved in DMSO (#D2650, Sigma-Aldrich) at a concentration of 10 mM. The stock solution was then diluted with cell culture medium to obtain 1000-µM, 100-µM, 10-µM and 1-µM solutions. *Trimipramine* (#T3146, Sigma-Aldrich) was dissolved in DMSO (#D2650, Sigma-Aldrich) at a concentration of 10 mM. The stock solution was then diluted with cell culture medium to obtain 10,000-µM, 1000-µM, 100-µM and 10-µM solutions. *Sirolimus* (#R0395, Sigma-Aldrich) was dissolved in DMSO (#D2650, Sigma-Aldrich) at a concentration of 10 mM. The stock solution was then diluted with cell culture medium to obtain 1000-µM, 100-µM, 10-µM and 1-µM solutions. The concentration range covered the therapeutic plasma concentrations of each treatment [desmethylclomipramine [37]; ionomycin [86]; trimipramine [87]; sirolimus [51]]. Drug-solution (or 3.5 µL cell culture media serving as vehicle) was added to cell culture media on the day of experiment (final incubation volume per well was 350 μL). As positive controls indicating a favourable outcome, we added either the inducible nitric oxide (NO) synthase inhibitor N-(3-(aminomethyl)benzyl)acetamidine (1400 W, 20 µM; #1415, Tocris, Bristol, UK) or anti-inflammatory cytokine interleukin 10 (IL-10, 50 ng/mL; #210-10, Peprotech, Rocky Hill, NJ, USA) into the four wells instead of the test compounds. Inflammation was induced by co-treatment with lipopolysaccharide (LPS; 200 ng/mL; #L5543, Sigma-Aldrich) and interferon-γ (IFNγ; 20 ng/mL; #I4777, Sigma-Aldrich) at 1 h after initiating the exposure to the test compounds. After 48 h, cell culture media was collected and stored −20 °C for the TNFα and nitrite concentration assays. Co-cultured cells were fixed to plates with 4% formaldehyde (#28908, Thermo Scientific) for 20 min at room temperature and stored at 4 °C until analysed in the cell viability assay. All experiments were performed twice, each including quadruplicates of each test compound and concentration.

#### 4.2.3. Effect of Test Compounds on In Vitro Monitoring Biomarkers with Focus on Inflammation, Nitric Oxide—Mediated Neurotoxicity and Neuronal Viability

*Neuroinflammation.* To assess the anti-inflammatory effects of the treatments, the level of secreted TNFα in the culture media was measured as a monitoring biomarker using an enzyme-linked immunosorbent assay (ELISA) kit (Ready-set-go mouse-TNFα, #88-7324-22; Affymetrix, Santa Clara, CA, USA) according to the manufacturer’s instructions. A 96-well plate was coated with capture antibody in 1X coating buffer overnight at 4 °C. The plate was washed three times and blocked with 1X ELISA/ELIPOT diluent in room temperature for 1 h. After washing the plate, 100 μL of culture media was added to the well and the plate was incubated overnight at 4 °C. A series of 2-fold standard dilutions was included in each plate. After incubation, the plate was washed three times, detection antibody was added and the plate was incubated for 30 min at room temperature and then the plate was washed three times. Avidin-HRP conjugate was added to the plate and incubated for 30 min at room temperature, after which the plate was washed five times. To quantify the peroxidase reaction, 1× TMB substrate solution was added to the plate for 30 min at room temperature, after which the reaction was stopped with 1M H_3_PO_4_. Absorbance was read at 450 nm and the TNFα concentration in the culture media was calculated from the standard curve using i-control software (Tecan Trading AG, Männedorf, Switzerland)

*Nitric oxide-mediated neurotoxicity.* To assess the effects of the treatments on nitric oxide-mediated neurotoxicity, nitrite (NO_2_^−^) levels in culture media formed by the spontaneous oxidation of NO were measured as a monitoring biomarker using Griess reagent (#G7921, Life Technologies, Carlsbad, CA, USA) according to the manufacturer’s instructions. Briefly, nitrite standards and a 1:5 dilution of each sample were added to a 96-well plate. A mixture containing equal volumes of component A and component B was added to each well of the plate and incubated for 30 min at room temperature, after which absorbance was measured at 548 nm. Nitrite concentrations in culture media were calculated from the standard curve in Excel 2016 (Microsoft, Redmond, WA, USA).

*Neuronal viability.* Neuronal viability was determined as previously described by Gresa-Arribas et al. [41]. Briefly, fixed co-cultures were rinsed twice with PBS. Cells were permeabilized with 0.3% H_2_O_2_ in methanol, after which they were blocked with 1% BSA and 10% horse serum in PBS for 20 min. Cells were immunostained overnight at 4 °C with anti-MAP2 monoclonal antibody (1:2000; #M9942, Sigma-Aldrich) that was diluted with blocking solution. The cells were then rinsed three times with PBS and incubated for 1 h at room temperature with biotinylated anti-mouse secondary antibody (1:500; #BA-2000, Vector Labs, Burlingame, CA, USA) diluted in blocking solution. Cells were washed three times and incubated with ExtrAvidin-HRP antibody (1:500; #E2886 Sigma-Aldrich) for 1 h at room temperature. The cells were then rinsed three times with PBS and incubated with 300 µL of ABTS peroxidase substrate (#SK-4500, Vector Labs) for 30 min according to the manufacturer’s instructions. Finally, 150 μL of substrate solution was transferred to a 96-well plate and absorbance was measured at 405 nm with an ELISA microplate reader (Tecan Infinite M200).

#### 4.2.4. Nfe2l2 Upregulation and Activation of Nrf2 Transcription Factor as Indicators of Target Engagement

Our earlier in silico analysis predicted upregulated gene expression of a transcription factor *Nfe2l2* by desmethylclomipramine and ionomycin [23], presumably leading to a favourable anti-oxidant effect on the basis of previous studies [52,88]. Therefore, we assessed the magnitude of the predicted upregulation of *Nfe2l2* gene and Nrf2 transcription factor target genes *Gclm*, *Hmox1* and *Nqo1* by test compounds in mouse BV2 microglia cell cultures under inflammatory and non-inflammatory conditions.

*BV2 microglia cell culture*. BV2 microglia were plated into 12-well plates (300,000 cells/well) and allowed to attach for 1 h in 1.5 mL of culture media. Desmethylclomipramine or ionomycin were dissolved in cell culture media, which was added to the plates at a final concentration of 1 µM (15 µL/well, total volume 1.5 mL). The concentration used was the most potent for reducing TNFα and nitrite formation in our prior co-culture experiments. Sulforaphane (#S-6317, Sigma-Aldrich), an Nrf2 activator [89], was added to the media as a positive control (final concentration 7.5 µM). After 2 h of BV2 plating, inflammation in desmethylclomipramine- and ionomycin-treated wells (*n* = 4 each) was induced with LPS (200 ng/mL; Sigma-Aldrich) and IFNγ (20 ng/mL; Sigma-Aldrich). An equal volume of culture medium was pipetted into control wells (*n* = 4). Another set of wells was treated similarly but not exposed to LPS and IFNy. After 16 h, the cell culture media was removed from each well and the cells were scraped into PBS for RNA extraction. The experiment was performed twice.

*RNA extraction.* RNA was extracted with NucleoZOL (#740404.200; Macherey&Nagel, Duren, Germany) according to the manufacturer’s instructions. Briefly, cells in PBS were centrifuged for 10 min (10,000× *g*) to pellet the cells and remove PBS, after which they were suspended in 500 µL of NucleoZOL. Then, 200 µL of water was added and the suspension was mixed vigorously and incubated for 15 min at room temperature. After a 15-min centrifugation (12,000× *g*) at 4 °C, the supernatant was collected to a new tube, 500 µL isopropanol was added and the mixture was incubated for 10 min at room temperature. After a 105-min centrifugation (12,000× *g*) at room temperature, the supernatant was discarded. The remaining pellet was washed with 500 µl of 75% ethanol and centrifuged (8000× *g*) twice for 3 min each. The pellet was dissolved in RNAase free water. The RNA concentration was quantified using a NanoDrop UV-VIS spectrophotometer.

*cDNA synthesis*. cDNA was synthesized with a Transcriptor First Strand cDNA Synthesis Kit (#04379012001, Roche, Basel, Switzerland) according to the manufacturer’s instructions. Briefly, 1 µg RNA and 1 µL Anchored-oligo(dT)_18_ primer in a total volume of 13 µL were incubated for 10 min at 65 °C with a PTC-100 thermal cycler. Then, 7 µL of reagent mix (4 µL Transcriptor Reverse Transcriptase Reaction Buffer, 0.5 µL Protector RNase Inhibitor, 2 µL Deoxynucleotide Mix and 0.5 µL Transcriptor Reverse Transcriptase) was added to the primer mix and the mixture was incubated for 30 min at 55 °C and for 5 min at 85 °C in a PTC-100 thermal cycler. The resulting cDNA was diluted 1:15 with water for quantitative polymerase chain reaction (PCR).

*Quantitative PCR.* Gene expression of *Nfe2l2*, *Gclm*, *Nqo1*, *Hmox1* and *Gapdh* in BV2 microglia culture was measured with Universal Probe Library System probes (Nfe2l2; #52, Gclm; #5, Nqo1; #50, Hmox1; #15, Gapdh; #77, #04683633001, Roche, Basel, Switzerland), using the following primer pairs (Nfe2l2-L; 5′TCATCCAGTTGAAACTGAGCAA-3′, Nfe2l2-R; 5′CGAAAAGGAAAGACAAGAGCA-3, Gclm-L; 5′GACTCACAATGACCCGAAAGA-3′, Gclm-R; 5′CTTCACGATGACCGAGTACCT-3′, Nqo1-L; 5′AGCGTTCGGTATTACGATCC-3′, Nqo1-R; 5′AGTACAATCAGGGCTCTTCTCG-3′, Hmox1-L; 5′GACACCTGAGGTCAAGCACA-3′, Hmox1-R; 5′ATCACCTGCAGCTCCTCAAA-3′, Gapdh-L; 5′AGCTTGTCATCAACGGGAAG-3′, Gapdh-R; 5′TTTGATGTTAGTGGGGTCTCG-‘3, Sigma-Aldrich) and FastStart Essential DNA Probes Master mix (#06924492001, Roche, Basel, Switzerland) according to the manufacturer’s instructions. Briefly, 5 µL master mix, 0.1 µL primers, 0.15 µL probe and cDNA corresponding to 3 µg RNA were added to a 20-µL reaction volume. Cycling (95 °C for 10 min, followed by 40 cycles of 95 °C for 10 s and 60 °C for 30 s) was conducted using a LightCycler 96 (Roche). Quantitation cycles were determined by the LightCycle 96 v1.1.0.1320 software (Roche). Gene expression levels were normalized against the *Gapdh* reference gene with the ΔCt-method.

#### 4.2.5. Scoring of Candidate Compounds for In Vivo Validation

To select compounds for further analysis, we generated a scoring system that takes into account: (a) the therapeutic time window, (b) pharmacokinetic properties (blood-brain barrier penetration and water solubility to allow administration via subcutaneous osmotic pumps), (c) anti-inflammatory, anti-oxidant and neuroprotective effects in vitro and (d) target engagement under non-inflammatory and inflammatory conditions. Scores varied from −3.6 to 4.6. The higher the score, the better the candidate for further analysis (Table 5). Desmethylclomipramine got the highest score (see Results and Table 4). Instead of administering desmethylclomipramine, however, we treated rats with clomipramine because it has good blood-brain barrier penetration [36,37] and is rapidly metabolized to desmethylclomipramine in vivo in both rodents and humans.

### 4.3. In Vivo Validation of Treatment on Post-TBI Functional Recovery

**Figure 6:**
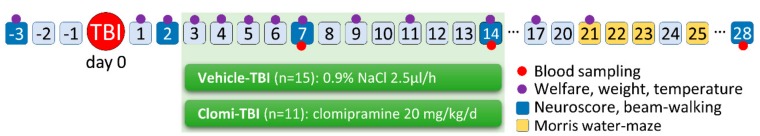
In vivo study design. Lateral fluid-percussion-induced (FPI) traumatic brain injury (TBI) was performed on day 0. Treatment with vehicle (Vehicle-TBI group, *n* = 15) or clomipramine (Clomi-TBI group, *n* = 11) was initiated between 08:00–12:00 at 3 days post-TBI via subcutaneous osmotic minipumps and discontinued between 08:00–12:00 at 14 days by removing the minipumps. Functional outcome was assessed by composite neuroscore and beam-walking tests before TBI (baseline) and at 2, 7, 14 and 28 days post-TBI. Spatial learning and memory were assessed with the Morris water-maze at 21–23 days and 25 days post-TBI. Tail vein blood was collected at 7, 14 and 28 days to assess plasma cytokine levels as monitoring biomarkers. General animal welfare was monitored 3 days before TBI and thereafter, at 1–7, 9, 11, 14, 17 and 21 days post-TBI. At the end of the experiment, rats were perfused for histology (see Figure S1).

In vivo study design is summarized in Figure 6.

#### 4.3.1. Animal Model and In Vivo Treatment Trial

*Induction of lateral fluid-percussion injury*. TBI was induced in 30 male Sprague-Dawley rats (age 13 weeks at the time of injury) with lateral FPI as previously described [90,91]. Briefly, the animals were anesthetized with a mixture of sodium pentobarbital (58 mg/kg), MgSO_4_*7H_2_O (127.2 mg/kg), propylene glycol (42.8%) and ethanol (11.6%) and administered intraperitoneally in a volume of 6 mL/kg. Once a surgical plane of anaesthesia was reached, the rats were placed into a stereotaxic frame with lambda and bregma at the same horizontal level. A 5-mm craniectomy was centred over the left cortex midway between lambda and bregma. A female Luer-Lock connector was placed into the craniotomy hole, its edges were carefully sealed with Vetbond tissue adhesive (3M, St. Paul, MN, USA) and the cap was surrounded with dental acrylate (Selectaplus powder #10009210; Selectaplus liquid CN #D10009102, DeguDent, Germany). Approximately 90 min after inducing anaesthesia, when the toe reflex reappeared, the rat was attached to the fluid-percussion device (AmScien Instruments, Richmond, VA, USA). The pendulum height was adjusted to produce severe injury (~3.0 atm; expected <48 h mortality 25%; Pitkänen and McIntosh (2006)) [92]. Post-impact seizure-like behaviour and apnoea duration were monitored. All animal procedures were approved by The Animal Ethics Committee of the Provincial Government of Southern Finland and performed in accordance with the guidelines of the European Community Council Directives 2010/63/EU.

*Post-impact monitoring and care*. After lateral FPI impact, the rats were placed on a heating pad (37 °C) until their toe reflex reappeared. Thereafter, they were placed in their home cage (1 rat/cage). For post-operative analgesia, the rats were treated with buprenorphine (0.05 mg/kg, s.c., Orion Pharma, Finland). To maintain fluid balance, they were injected with 10 mL of 0.9% NaCl (twice a day, s.c.) for 3 d. To aid feeding, a powdered pellet (Teklad 2016S, Envigo) diet was available for the first 3 days after injury or until the animals were able to eat solid pellets and drink on their own.

*Randomization of animals to different treatment groups.* Rats (*n* = 30) underwent lateral FPI. Twenty-six animals survived and were randomized to two treatment groups at 3 days post-TBI: (1) Vehicle-TBI group (*n* = 15) and (2) clomipramine-TBI (Clomi-TBI) group (*n* = 11).

*Clomipramine administration.* To modulate the neuroinflammation and neurodegeneration by clomipramine within the time window of target expression, the 11-day long treatment was started in the morning at 3 days post-TBI and the minipump was removed 14 days post-TBI (Figure 6). Because the half-life of clomipramine in rats is approximately 4 h, it was administered via subcutaneous osmotic minipumps to maintain a constant therapeutic concentration [93,94].

Clomipramine (#C7291, Sigma-Aldrich) was dissolved in sterile 0.9% NaCl (25 mg/mL) and administered at a dose of 20 mg/kg/d (Alzet 2ML1, Alzet, DURECT Corporation, Cupertino, CA, USA). Osmotic pumps were filled and used as described by the manufacturer. Briefly, an empty pump was weighed, filled with drug solution using a blunt-end needle and weighed again to confirm that the pump was filled. The pumps were then incubated overnight in sterile 0.9% NaCl at 37 °C to ensure drug-delivery from the pumps immediately after subcutaneous implantation. To subcutaneously implant the pumps into the interscapular space in the back, anaesthesia was induced with 4% isoflurane inhalation in a transparent plastic chamber. Anaesthesia was maintained with 2.0% isoflurane inhalation with a nose mask. The clomipramine-filled pumps were replaced with new pumps at 7 days after implantation under isoflurane anaesthesia.

*Vehicle administration*. Sterile 0.9% NaCl was administered for 11 days at 2.5 µL/h via subcutaneous minipumps (Alzet 2ML4).

All pumps were removed under anaesthesia in the morning at 14 days post-TBI and inspected to confirm that the contents had been delivered.

#### 4.3.2. Behavioural Analysis

*Composite neuroscore.* The neuroscore is a sensitive indicator of the effect of pharmacologic manipulations [90,95]. To evaluate the treatment effect on somatomotor recovery, we assessed the neuroscore before TBI (baseline) and at 2, 7, 14and 28 days post-TBI. Animals were scored from 0 (severely impaired) to 4 (normal) for each of the following 7 indices: (a) left and right (2 indices) forelimb flexion during tail suspension, (b) left and right (2 indices) hindlimb flexion when the forelimbs remained on a hard surface and the hindlimbs were lifted up and back by the tail, (c) ability to resist a lateral pulsion toward the left and right (2 indices) and (d) angle board. The details of the procedure are described in Nissinen et al. (2017) [96].

*Beam-walking.* To evaluate coordination and motor movement, beam-walking was tested before TBI (baseline) and at 2, 7, 14and 28 days post-TBI, as described in Nissinen et al. (2017) [96]. The beam was a 139-cm long and 2.1-cm wide wooden bar with a black box (25 cm × 20 cm) at the right end of the beam. The beam was placed 43 cm above the floor next to a mirror, 30 cm from the wall. The rats were trained to cross the beam 1 day before baseline testing. In the training, the rat was first placed into the box for 1 min. Thereafter, the rat was placed on the beam at a gradually longer (15, 35, 70 and 100 cm) distance from the box and allowed to walk into the box and stay there for 1 min. Training was completed when the rat quickly crossed the whole beam. On testing days, three trials were conducted and between each trial the rat remained in the box for 1 min. The trials were scored from 0 (rat fell down) to 6 (rat crossed the beam with no foot slips). Scoring was as follows: Score 0: rat fell down. Score 1: rat was unable to traverse the beam but remained sitting across the beam. Score 2: rat fell down while walking. Score 3: rat could traverse the beam but the affected hind limb did not aid in forward movement. Score 4: rat traversed the beam with more than 50% foot slips. Score 5: rat crossed the beam with 50% or fewer foot slips. Score 6: rat crossed the beam with no foot slips. The mean score of the three trials was calculated.

*Morris water maze.* Spatial learning and memory performance were tested in the Morris water-maze using a 5-d paradigm at 21–25 days post-TBI (Figure 6). The test was performed in a round 150-cm diameter pool. Water temperature was maintained at 20 ± 0.5 °C. The submerged escape platform (1 cm below water surface, size 10 cm × 10 cm) was in the northeast quadrant. Starting position of the trials varied among the four constant locations at the pool rim (North, East, South, West), so that all rats started from the same position in any single trial. If the rat failed to find the escape platform within 60 s, it was placed on the platform for 10 s by the experimenter. Between trials, rats were given a 1-min recovery period. The rats were given five trials/day on days 1 and 2. On day 3, five trials were given, followed by a probe trial without a platform and then a trial with a platform. On the day 4, no trials were conducted. On day 5, a probe trial without a platform was performed. Trials were video-recorded with a camera mounted on the ceiling. Swimming speed, path length, time spent in the platform quadrant and latency to reach the platform were measured for each trial with EthoVision XT software (Noldus, Wageningen, The Netherlands).

#### 4.3.3. Monitoring of Adverse Events

*General health.* The general health of the rats was monitored before TBI (once between 4–6 days pre-TBI, baseline) and once per day at 1–7, 9, 11, 14, 17 and 21 days post-TBI, as described previously [97]. Health monitoring included checking for external bleeding (mouth, nose, anus, eyes and toes), evaluation of pain (vocalization, hunched and elevated posture, grooming, withdrawn, dystonia and self-mutilation), fur condition (barbering, hair loss, scratching, rash, dirty hair), bowel function (faecal stains, diarrhoea and constipation), motor function (hindlimb clasping, limping, ataxia or other mobility problems) and body condition scoring [98].

*Rectal temperature and body weight.* Rectal temperature and body weight were monitored before TBI (once at 4–6 days pre-TBI, baseline) and once per day at 1–7, 9, 11, 14, 17 and 21 days post-TBI.

#### 4.3.4. Histology

Rats were perfused for histology at 28 days post-TBI to assess the location and extent of cortical lesion as described previously [99]. Briefly, rats were deeply anesthetized and perfused with 0.9% NaCl solution (2 min, 3 mL/min), followed by cold (4 °C) 4% paraformaldehyde (30 min, 3 mL/min). The brains were removed from the skull, post-fixed in 4% PFA for 2 h at 4 °C and then cryoprotected in 20% glycerol prepared in 0.02 M potassium phosphate buffer solution for 48 h at 4 °C. The brains were frozen on dry ice and stored at −70 °C until further processing. The brains were cut into 30-µm-thick coronal sections (1-in-5 series) using a sliding microtome. The first series of sections was used for thionin staining to assess the tissue cytoarchitectonics and lesion location and extent.

#### 4.3.5. Analysis of Plasma-Monitored Biomarkers

*Blood sampling and preparation of plasma.* Blood was sampled from the tail vein at 7, 14 and 28 days post-TBI (Figure 6). The blood sampling procedure was described previously in van Vliet EA et al. [100]. Briefly, the rats were lightly sedated with isoflurane. Then, 2 × 0.5 mL of blood were collected from the distal one-third of the tail with a 23G butterfly needle (#367284, BD, Franklin Lakes, NJ, USA) into EDTA tubes (#365975, BD). To mix the blood and EDTA, the tube was inverted 10 times. To separate the plasma, the blood was centrifuged at 1300× *g* for 10 min at 4 °C. To quantify haemolysis, haemoglobin absorbance was measured at 414 nm from 1.5 µL of plasma with Nanodrop-1000 UV-VIS spectrophotometer (Thermo Fisher Scientific). Plasma was aliquoted to micro-centrifuge tubes (50 µL; #022431064, Eppendorf, Hamburg, Germany) and kept −70 °C for further analysis.

*Cytokine assay*. A total of 12 cytokines (GM-CSF, IFNy, IL-1α, IL-1β, IL-2, IL-4, IL-5, IL-6, IL-10, IL-12p70, IL-13 and TNFα) were measured from the plasma of Vehicle-TBI and Clomi-TBI treated rats sampled at 7, 14 and 28 days post-TBI, using Bio-Plex Pro Rat Cytokine Th1/Th2 panel (#171K1002M, BioRad, Hercules, CA, USA) with the Bio-Plex 200 System (BioRad), according to the manufacturer’s instructions. Briefly, 30 µL of rat plasma was diluted 1:4 with Bio-Plex sample diluent. Then, 50 µL of diluted duplicate plasma sample and assay standard (1 per plate) were pipetted into a 96-well assay plate. Magnetic beads (50 μL) were added to each well and the plate was placed on a microplate shaker (850 rpm, Thermo Scientific, MiniMix MN201) for 1 h at room temperature. After incubation, the beads were washed three times with Bio-Plex wash buffer using a Handheld Magnetic Washer (BioRad, #171020100). Detection antibody was added and incubation was continued for 30 min. After three washes, SA-PE conjugate was added to the wells and incubation was continued for 10 min. After three washes, magnetic beads containing the cytokines were resuspended to a 125-µL assay buffer on a shaking plate (30 s, 850 rpm). Finally, the fluorescence of the beads was quantified using the Bio-Plex 200 System. Cytokine concentrations in each sample were calculated using Bio-Plex 4.1.1 software (BioRad).

#### 4.3.6. Statistics

*Co-culture experiments.* Effect of a given drug on TNFα, NO and neuronal viability (4 wells/concentration/drug, two independent experiments, resulting in a total of eight data points per treatment) was compared to vehicle-treated controls with Mann-Whitney U-test in R (R version 3.3, Wilcox-test function)[101].

*BV2 microglia experiments.* The effect of a given treatment on the expression levels of *Nfe2l2*, *Gclm*, *Hmox1* and *Nqo1* in the 16-h incubation experiment performed in duplicate was analysed using a linear regression model (normalizing batch effect) in R (R version 3.3, lm function). The single 48-h experiment was analysed with a linear regression model.

*In vivo experiments*. Composite neuroscore, beam-walking, plasma cytokine concentrations, rectal temperature and body weight were analysed with a linear mixed effects model, consisting of the systematic and random parts (R version 3.5, lme function). The systematic part in the model contains fixed effects, time and drug. The random part of the model takes into account the structural dependency between measurements that arises from repeated measurements on animals and grouping of animals. The model itself was as follows:

*y* = *XB* + *Zu* + *ε* (1)

where *y* is a *N* × 1 column vector, the outcome variable; *X* is a *N* × *p* matrix of the *p* predictor variables; *B* is a *p* × 1 column vector of the fixed-effects regression coefficients; *Z* is the *N* × *q* design matrix for the *q* random effects; *u* is a *q* × 1 vector of the random effects (the random complement to the fixed *B*); and *ε* is a *N* × 1 column vector of the residuals, that part of *y* that is not explained by the model. *p*-values were adjusted with Bonferroni’s correction for multiple comparisons. Performance in the Morris water-maze test was analysed using the Cox proportional hazards model (R version 3.5, coxph and coxme functions) with the individual frailty of each animal using the latency of the 5th swim at 21–23 days post-TBI. The differences in the post-TBI probe trials at 23 and 25 days was tested with the Kruskal-Wallis test (R version 3.3, kruskal.test function). Animal health between the treatment groups was analysed with Pearson’s Chi-squared test (R.version 3.3, chisq.test function). All statistical analyses were considered statistically significant when the *p*-value was less than 0.05.

*Machine learning (ML) analysis of the treatment effects on the plasma cytokine levels and* in vivo *outcome measures***.** The ML models utilized in classification of treatment effects with radial gradient boosted trees [102,103]. The feature sets included in the analysis are summarized in Table S5. For all feature sets, excluding cytokine measurements from individual days, differences between subsequent measurements and the slope of a line fitted through all the measurements in the series were included as additional features.

Model performance evaluation and hyperparameter optimization was performed via nested cross-validation (CV) with grid search [104,105], using a leave-one-out CV on the outer and stratified 10-fold split in the inner level of the search. In the nested CV grid search, the dataset was split into outer-testing and outer-training sets. The outer-training sets were further split into inner-training and inner-validation sets. The classifiers were trained using the inner-training sets and evaluated on the inner-validation sets for each possible combination of hyperparameters. The hyperparameters resulting in the best performance in terms of area under the receiver operating characteristic curve (AUC)-score within the outer-training set were used for classification in the outer-testing set.

Feature selection by univariate filtering, embedding and dimensionality reduction was included in the inner cross-validation loop. In the univariate filtering, the features were ranked according to the F-score and the percentile of the highest-ranking features selected was varied during the grid search. In embedding, the features were extracted from an extreme random forest classifier [106] trained on the inner-training data. Features with above average weight in extreme random forest were extracted as wrapped features. Dimensionality reduction was performed using principal component analysis. For the cytokine measurements at 7, 14 and 28 days, the feature selection step was omitted.

During the cross-validation, all variables were standardized to zero mean and unit variance. Standardization of the test set was performed using the mean and standard deviation of the training set to avoid bias by information leakage from the training to testing.

Classification performance was evaluated in terms of the AUC, which was calculated over the pooled predictions on the outer test set. Pairs of classifiers and feature sets that attained an AUC > 0.75 during cross-validation were selected for further study. For the selected classifiers, CV with a nested grid search was repeated 10 times with different random classifier initialization and stratified 10-fold splits. This is to prevent bias caused by specific random classifier initialization or split of data to CV folds. When the cross-validation AUC for a classification and feature set pair was above 0.75, permutation testing was performed to evaluate the statistical significance of the classification score [107].

## 5. Conclusions

Currently, there are no treatments available to combat post-TBI secondary damage and improve functional outcome [3,108,109,110]. Traditionally, drug discovery and testing for TBI treatments have relied on a hypothesis-driven approach (see Kochanek et al., 2011) [111]. Here, we introduce a novel non-hypothesis based in silico analysis-driven approach that aims to reverse/augment the regulated transcriptomics gene networks with an ultimate goal to improve recovery. We designed an in silico in vitro → in vivo pipeline and scoring for stepwise assessment of candidate compounds with a → focus on compounds that can be repurposed for TBI to hasten clinical development. Desmethylclomipramine, a widely used antidepressant, was taken through the pipeline all the way to proof-of-concept in vivo testing. Despite favourable in silico and in vitro outcomes, in vivo assessment of clomipramine, which is rapidly metabolized to desmethylclomipramine, demonstrated no favourable effects. Rather, clomipramine treatment in the subacute post-TBI phase prolonged the elevation of plasma inflammatory monitoring biomarkers. It is possible that weight-loss–related stress contributed to the prolonged upregulation of plasma cytokines and even the negative functional outcome, which emphasizes the need for adverse event monitoring in preclinical studies. Taken together, our pipeline provides a rational stepwise procedure for evaluating the effects of systems-biology–discovered compounds on post-TBI transcriptomics. The sensitivity and specificity of the pipeline can be improved by tailoring the number and type of in vitro monitoring biomarkers and taking account known side effects of the compounds. Also, refinement of the scoring system used to compare compounds and establish the threshold for in vivo analysis will likely increase the objectivity and performance of the pipeline which we are continuously improving.

## Figures and Tables

**Table 1:** Concordance value, cell line from which the data were derived and signature ID in the iLINCS analysis. Gene expression signatures were assessed in the expression datasets of the perilesional cortex obtained at 32 h and 3 months post-TBI.

Drug	32 H			3 Months		
	Concordance	Cell Line	Signature ID	Concordance	Cell Line	Signature ID
Desmethylclomipramine	0.123248134	NEU.KCL	LINCSCP_40633	0.239423	NEU.KCL	LINCSCP_40633
Ionomycin	0.11614885	NEU	LINCSCP_41477	0.225506	NEU.KCL	LINCSCP_40564
Trimipramine	0.106234243	NEU	LINCSCP_40955	No score	No score	No score
Sirolimus	No score	No score	No score	−0.18388	NPC	LINCSCP_42468

**Table 2:** Gene expression of *Nfe2l2* and Nrf2 target genes, nitric oxide synthase and cytokine-encoding genes at 32 h and 3 months post-TBI in the rat perilesional cortex.

Gene	32 H		3 Months	
	Log2 FC	FDR	Log2 FC	FDR
***Nfe2l2* and Nrf2 Target Gene Expression**				
*Nfe2l2*	0.094	6.41 × 10^−1^	**0.601**	**6.41** **× 10^−5^**
*Gclm*	**0.818**	**1.88** **× 10^−8^**	0.016	9.51 × 10^−1^
*Hmox1*	**2.860**	**1.69** **× 10^−9^**	**1.189**	**2.69** **× 10^−18^**
*Nqo1*	**0.424**	**6.45 × 10^−3^**	**0.646**	**1.29** **× 10^−7^**
**Nitric Oxide Synthase Gene Expression**				
*Nos1*	0.038	6.22 × 10^−1^	0.209	3.50 × 10^−1^
*Nos2*	**−0.324**	**7.05** **× 10^−7^**	**0.587**	**3.32 × 10^−2^**
*Nos3*	0.203	1.22 × 10^−1^	−0.023	9.30 × 10^−1^
**Cytokine Gene Expression**				
*Il1a*	**−0.693**	**1.08 × 10^−4^**	−0.089	8.50 × 10^−1^
*Il1b*	**0.478**	**6.15 × 10^−2^**	0.598	NA
*Il2*	**−0.314**	**6.57** **× 10^−6^**	−0.009	NA
*Il4*	**−0.177**	**1.38 × 10^−4^**	0.095	NA
*Il5*	**−0.149**	**9.87× 10^−4^**	NA	NA
*Il6*	**0.409**	**2.62 × 10^−2^**	−0.013	NA
*Il10*	**−0.300**	**3.81 × 10^−3^**	0.161	NA
*Il12a*	**−0.409**	**5.12** **× 10^−5^**	−0.002	9.96 × 10^−1^
*IL-13*	**−0.247**	**1.39 × 10^−3^**	−0.046	NA
*Csf2* (GM-CSF)	**−0.211**	**5.91 × 10^−3^**	NA	NA
*Ifng*	**−0.414**	**2.19 × 10^−2^**	NA	NA
*Tnf*	−0.147	5.24 × 10^−1^	0.088	8.53 × 10^−1^

Abbreviations: FC, fold-change as compared injured to sham-operated experimental controls; FDR, false discovery rate; NA, no detectable expression or no *p*-value available. At 32 h post-TBI, 3 sham-operated and 3 injured controls were used. At 3 months post-TBI, 5 sham-operated and 5 injured rats were used. Statistical significance: FC and corresponding FDR (<0.05) are shown in bold font.

**Table 3:** Target engagements measured as a gene expression of *Nfe2l2*, *Gclm*, *Hmox1 and Nqo1* after 16-h treatment with desmethylclomipramine, ionomycin or sulforaphane under LPS/IFNy-induced inflammatory or non-inflammatory conditions in BV2-microglia cell cultures.

Drug	Inflammatory (LPS/IFNy)		Non-Inflammatory (No LPS/IFNy)	
	Log2 FC	*p*-Value	Log2 FC	*p*-Value
**Desmethylclomipramine**				
*Nfe2l2*	**0.282**	**1.87 × 10^−2^**	**0.200**	**3.83 × 10^−2^**
*Gclm*	0.099	7.70 × 10^−1^	0.118	7.83 × 10^−1^
*Hmox1*	0.039	7.85 × 10^−1^	0.050	3.98 × 10^−1^
*Nqo1*	−0.056	6.64 × 10^−1^	−12.419	1.09 × 10^−1^
**Ionomycin**				
*Nfe2l2*	−0.052	7.87 × 10^−1^	0.125	1.65 × 10^−1^
*Gclm*	0.262	1.04 × 10^−1^	0.388	4.52 × 10^−1^
*Hmox1*	0.298	1.54 × 10^-1^	0.281	6.58 × 10^-2^
*Nqo1*	**0.565**	**8.72 × 10^-3^**	−12.283	8.89 × 10^-2^
**Sulforaphane**				
*Nfe2l2*	**0.723**	**3.01** **× 10^−7^**	**−0.382**	**1.26 × 10^-2^**
*Gclm*	**1.117**	**7.47** **× 10^−8^**	**3.305**	**4.61** **× 10^−15^**
*Hmox1*	**0.171**	**1.52 × 10^-2^**	**1.403**	**3.14** **× 10^−9^**
*Nqo1*	**1.897**	**1.03** **× 10^−12^**	−6.055	7.23 × 10^-2^

Abbreviations: FC, fold-change compared to untreated control; LPS, lipopolysaccharide, IFNγ, interferon gamma. The experiment was performed twice, each including quadruplicates. Statistical significances: FC-change and corresponding *p*-value (<0.05) from linear regression model are shown in bold font.

**Table 4:** Compound performance scores based on in silico and in vitro validation.

Scoring Component	Desmethylclomipramine	Ionomycin	Trimipramine	Sirolimus	1400W	IL-10
**In Silico Analysis of Therapeutic Time Window**						
Concordance (acute)	0	0.3	0	No score	No score	No score
Concordance (chronic)	0.3	0.3	0	0	No score	No score
**Pharmacokinetic Properties**						
Blood brain barrier penetration	0.5	−0.5	0.5	0.5	0.5	0
Water solubility	0.5	0	0.5	0.5	0.5	0.5
Subtotal	**1.3**	**−0.2**	**1**	**1**	**1**	**0.5**
**Co-culture Outcome**						
Inflammation (TNFα)	0.3	0.3	0.3	0.3	0.6	0.6
Oxidative stress (NO)	0.15	0.15	0.15	0.3	0.15	0.15
Neuronal viability (MAP2)	0	0	0	0.2	0.4	0.4
Subtotal	**1.75**	**0.25**	**1.45**	**1.8**	**1.15**	**1.65**
**Target Engagement (Inflammatory Condition)**						
*Nfe2l2*	0.0125	0				
*Gclm*	0	0				
*Hmox1*	0	0				
*Nqo1*	0	0.0125				
**Target Engagement (Non-inflammatory Condition)**						
*Nfe2l2*	0.0125	0				
*Gclm*	0	0				
*Hmox1*	0	0				
*Nqo1*	0	0				
Total Score	**1.775**	**0.2625**				

Abbreviations: IL, interleukin; N-(3-(aminomethyl) benzyl) acetamidine, 1400 W (inducible nitric oxide synthase inhibitor); MAP2, microtubule-associated protein 2; NO, nitric oxide; TNFα, tumour necrosis factor alpha. Subtotal (bold number) is the sum scores from beginning of the column.

**Table 5:** Weighted scoring of compound performance based on in silico and in vitro analysis to facilitate compound selection for in vivo validation.

Scoring Component	−5	−1	0	1	2	3	4	5	Weight
**In Silico Analysis of Therapeutic Time Window**									
Concordance (acute)			>Top 60% ^#^	Top 50% ^#^	Top 40% ^#^	Top 30% ^#^	Top 20% ^#^	Top 10% ^#^	10%
Concordance (chronic)			>Top 60% ^#^	Top 50% ^#^	Top 40% ^#^	Top 30% ^#^	Top 20% ^#^	Top 10% ^#^	10%
**Pharmacokinetic Properties**									
Blood brain barrier penetration	No		Unknown					Yes	10%
Water solubility	Non soluble		Unknown					Soluble	10%
**Co-culture Outcome**									
Inflammation (TNFa)	>100%^&^		100% ^&^	<90% ^&^	<70% ^&^	<50% ^&^	<30% ^&^	<10% ^&^	15%
Neurotoxicity (NO)	>100%^&^		200% ^&^	<90% ^&^	<70% ^&^	<50% ^&^	<30% ^&^	<10% ^&^	15%
Neuronal viability (MAP2)	<100%^&^		>115% ^&^	>100% ^&^	>150% ^&^	>200% ^&^	>250% ^&^	>300% ^&^	20%
**Target Engagement (under Inflammatory Conditions)**									
*Nfe2l2*		↓	-	↑					1.25%
*Gclm*		↓	-	↑					1.25%
*Hmox1*		↓	-	↑					1.25%
*Nqo1*		↓	-	↑					1.25%
**Target Engagement (under Non-inflammatory Condition)**									
*Nfe2l2*		↓	-	↑					1.25%
*Gclm*		↓	-	↑					1.25%
*Hmox1*		↓	-	↑					1.25%
*Nqo1*		↓	-	↑					1.25%

The highest achievable score was 4.6 and the lowest −3.6. The higher the score, the more effective the candidate compound. Abbreviations: In silico analysis: #, compounds were arranged according to the absolute value of the iLINCS concordance score. The compounds with a concordance score in the top 10% received 5 points, in the top 10–20%, 4 points, etc.; Co-culture outcome: &, percentage of the effect compared with the non-treated control; MAP2, microtubule-associated protein 2; NO, nitric oxide; TNFα, tumour necrosis factor alpha. Target engagement: ↓, statistically significant downregulation in gene expression compared with the non-treated control; -, no effect on gene expression; ↑, statistically significant upregulation in gene expression compared with the non-treated control.

## Supplementary Materials

The following are available online at https://www.mdpi.com/1422-0067/20/21/5395/s1, Supplementary Tables 1–5 and Supplementary Figure S1.

## Abbreviations

AUC	area under the curve
CMap	Connectivity Map
DMEM	Dulbecco’s modified Eagles medium
DMSO	dimethyl sulfoxide
ELISA	enzyme-linked immunosorbent assay
FBS	foetal bovine serum
FDR	false discovery rate
FPI	fluid-percussion injury
GM-CSF	granulocyte-macrophage colony-stimulating factor
IFNy	interferon gamma
IL	interleukin
LINCS	Library of Integrated Network-Based Cellular Signatures
LPS	lipopolysaccharide
MAP2	microtubule-associated protein 2
ML	machine learning
NO	nitric oxide
NO_2-_	nitrite
PBS	phosphate-buffered saline
STAR	Spliced Transcripts Alignment to Reference
TBI	traumatic brain injury
TNFα	tumour necrosis factor alpha
1400W	N-(3-(aminomethyl)benzyl)acetamidine
