# Supplementary material for: In Vitro and In Vivo Pipeline for Validation of Disease-Modifying Effects of Systems Biology-Derived Network Treatments for Traumatic Brain Injury—Lessons Learned
Source: Int J Mol Sci. 2019 Oct 29;20(21):5395. doi: 10.3390/ijms20215395 (PMC6861918; doi:10.3390/ijms20215395)
Supplement: Supplementary file 1 [file ijms-20-05395-s001.pdf]

In vitro and in vivo pipeline for validation of disease-modifying effects of systems biology-derived network treatments for traumatic brain injury – lessons learned

Anssi Lipponen, Teemu Natunen, Mika Hujo, Robert Ciszek, Elina Hämäläinen, Jussi Tohka, Mikko Hiltunen, Jussi Paananen, David Poulsen, Emilia Kansanen, Xavier Ekolle Ndode-Ekane, Anna-Liisa Levonen and Asla Pitkänen.

Table S1. Effect of test compounds (desmethylclomipramine, ionomycin, sirolimus, trimipramine) and positive controls (1400W, IL-10) on TNFα and nitrite concentrations and neuronal viability in neuron-BV2 microglia co-cultures after 48-h drug treatment.

	**TNFα**			**Nitrite**			**Neuronal viability**		
**Drug**	**% of control**	**Mean ± SEM pg/ml**	**p-value**	**% of control**	**Mean ± SEM µM**	**p-value**	**% of control**	**Mean ± SEM AU**	**p-value**
**Desmethylclomipramine**									
10 µM	-	-	-	-	-	-	-	-	-
1 µM	**71.7 %**	**492.1 ± 32.5**	**4.451e-5**	**85.2 %**	**72.3 ± 2.7**	**0.0005953**	114.2 %	0.1793 ± 0.012	0.08836
0.1 µM	**74.5 %**	**511.4 ± 36.0**	**0.000825**	**86.1 %**	**73.1 ± 2.7**	**0.002643**	102.6 %	0.1611 ± 0.009	0.6913
0.01 µM	88.4 %	607.0 ± 33.9	0.133	96.7 %	82.0 ± 2.9	0.8706	93.4 %	0.1466 ± 0.007	0.323
**Ionomycin**									
10 µM	-	-	-	-	-	-	-	-	-
1 µM	**55.8 %**	**382.8 ± 12.4**	**2.71e-6**	**79.8 %**	**67.8 ± 2.1**	**6.764e-5**	123.3 %	0.1936 ± 0.022	0.2126
0.1 µM	**59.3 %**	**406.8 ± 16.9**	**2.719e-6**	**82.0 %**	**69.6 ± 1.4**	**0.001602**	113.0 %	0.1774 ± 0.015	0.2931
0.01 µM	**70.9 %**	**486.5 ± 29.5**	**0.0005112**	**81.7 %**	**69.4 ± 2.2**	**0.0005116**	108.9 %	0.1709 ± 0.010	0.2509
**Sirolimus**									
10 µM	-	-	-	-	-	-	-	-	-
1 µM	**60.7 %**	**416.4 ± 26.4**	**3.263e-5**	**63.1 %**	**53.5 ± 3.0**	**1.59e-7**	**144.7 %**	**0.2272 ± 0.019**	**0.0004456**
0.1 µM	**61.2 %**	**420.1 ± 29.1**	**5.167e-5**	**66.0 %**	**56.0 ± 3.1**	**2.385e-7**	**133.4 %**	**0.2094 ± 0.019**	**0.01123**
0.01 µM	**68.2 %**	**468.1 ± 38.4**	**0.0005112**	**78.9 %**	**66.9 ± 4.3**	**0.0018**	110.8 %	0.1740 ± 0.013	0.2338
**Trimipramine**									
100 µM	-	-	-	-	-	-	-	-	-
10 µM	**61.2 %**	**420.3 ± 28.3**	**5.439e-6**	**76.9 %**	**65.2 ± 2.5**	**4.695e-5**	104.2 %	0.1636 ± 0.012	0.6153
1 µM	**69.9 %**	**479.5 ± 24.7**	**5.167e-6**	**83.7 %**	**71.0 ± 1.2**	**0.0007629**	106.3 %	0.1668 ± 0.009	0.3058
0.1 µM	**77.5 %**	**532.2 ± 27.0**	**0.004601**	**88.2 %**	**74.9 ± 2.0**	**0.02164**	102.7 %	0.1612 ± 0.010	0.8111
**1400W**									
20 µM	102.6 %	768.1 ± 45.2	0.9591	**5.7 %**	**4.8 ± 1.1**	**1.519e-5**	**257.1 %**	**0.4036 ± 0.023**	**4.445e-5**
**IL10**									
50 ng/ml	**26.9 %**	**161.2 ± 26.9**	**2.179e-6**	**68.8 %**	**68.5 ± 1.6**	**1.679e-6**	**149.8 %**	**0.2351 ± 0.013**	**7.114e-7**

Abbreviations: AU, absorbance unit; SEM, standard error of mean; -, toxic concentration. ***Statistical significance:*** p-values (<0.05, Mann-Whitney *U* test) and corresponding concentrations are shown in bold font.

**Table S2.** Risk factors given by the Cox proportional hazard model for latency to find the platform in the Morris water-maze test. Interaction model between the latency and testing day was constructed to calculate risk factors for finding the platform. A risk factor >1 indicates that that "the risk of finding a submerged platform" was higher in the Clomi-TBI group than in the vehicle-TBI group (*i.e.,* Clomi-TBI animals exhibited improved performance).	
	**Clomi-TBI**
**Day 1**	0.5088001
**Day 2**	0.8252243
**Day 3**	1.413685
Model was not statistically significant (p= 0.5351). Abbreviations: Clomi, clomipramine; TBI, traumatic brain injury.	

Table S3. Plasma cytokine concentrations in the Vehicle-TBI and Clomi-TBI groups at 7 d, 14 d, and 28 d after TBI. Mean concentration, confidence intervals and p-values are from linear mixed effects model. Cytokine concentration in the Vehicle-TBI and Clomi-TBI groups did not differ at 7 d, 14 d, and 20 d post-TBI (column 7-28 d). Concentrations of plasma cytokines were reduced at 28 d compared with 7 d.

	**7-28 d**	**7 d**		**14 d**			**28 d**		
**Cytokine**	**Vehicle-TBI vs. Clomi-TBI p-value**	**Mean [CI lower, upper] pg/ml**		**Mean [CI lower, upper] pg/ml**		**7 d vs. 14 d p-value**	**Mean [CI lower, upper] pg/ml**		**7 d vs. 28 d p-value**
		**Vehicle-TBI**	**Clomi-TBI**	**Vehicle-TBI**	**Clomi-TBI**		**Vehicle-TBI**	**Clomi-TBI**	
GM-CSF	0.2195	62.7 [45.6, 79.7]	43.8 [28.7, 58.9]	52.4 [37.4, 66.8]	56.2 [37.3, 75.2]	0.9832	18.3 [-0.19, 36.7]	38.8 [16.2, 61.5]	0.1009
IL-1α	0.392	229.9 [119.5, 340.3]	215.4 [96.1, 334.1]	218.3 [110.2, 326.2]	178.02 [68.5, 287.6]	0.6497	46.2 [-40.6, 132.9]	64.2 [-24.5, 152.9]	**<0.0001**
IL-1β	0.476	145.8 [78.0, 213.4]	133.2 [62.0, 204.4]	149.0 [79.4, 218.5]	123.7 [53.3, 194.1]	0.9895	28.6 [-23.8, 81.0]	41.7 [-12.5, 96.0]	**<0.0001**
IL-4	0.204	140.7 [86.6, 194.8]	126.9 [68.2, 185.5]	156.3 [97.8, 214.8]	124.7 [63.8, 185.5]	0.4846	21.7 [-13.8, 57.1]	65.6 [12.4, 118.8]	**0.00005**
IL-5	0.5285	670.3 [439.1, 901.4]	698.4 [453.1, 943.8]	762.5 [528.8, 996.3]	661.7 [416.3, 907.1]	0.5843	290.4 [40.4, 540.3]	405.9 [148.8, 663.1]	**<0.0001**
IL-6	0.3445	1419.9 [837.1, 2002.7]	1370.4 [739.5, 2001.2]	1633.1 [1021.0, 2245.2]	1364 [712.9, 2016.4]	0.3775	366.9 [-141.0, 874.7]	806.3 [156.3, 1456.4]	**0.0024**
IL-10	0.2418	243.8 [132.3, 355.2]	229.3 [109.2, 349.5]	241.6 [130.6, 352.7]	189.3 [78.3, 300.3]	0.7996	52.7 [-36.4, 141.6]	89.9 [-5.6, 185.3]	**<0.0001**
IL-12p70	0.5300	818.7 [431.6, 1205.8]	717.6 [307.8, 1127.4]	893.1 [482.6, 1303.5]	689.9 [270.7, 1109.2]	0.4894	144.5[-131.9, 446.0]	204.5 [-91.4, 500.4]	**0.0001**
IL-13	0.5095	728.0 [512.9, 943.1]	680.7 [443.6, 917.7]	817.9 [594.1, 1041.7]	753.5 [479.4, 1027.6]	0.3876	230.1 [39.6, 420.6]	470.7[180.8, 760.7]	**0.0036**
IL-2	0.8073	5856.4 [4374.2, 7338.6]	5772.5 [4018.7, 7526.3]	6474.8 [4936.6, 8013.0]	5750.6 [3902.0, 7599.3]	0.6597	1574.1 [-522.1, 3670.2]	2728.1 [632.5, 4824.7]	**0.0002**
IFNy	0.1902	355.0 [204.0, 506.0]	316.6 [154.2, 479.1]	372.3 [217.7, 527.0]	261.2 [113.7, 408.7]	0.8536	64.0 [-37.1, 165.1]	166.3 [19.3, 313.2]	**<0.0001**
TNFα	0.7122	1342 [1020.0, 1664.3]	1432.0 [1050.8, 1813.2]	1505.2 [1170.9, 1839.6]	1446.9 [1045.1, 1848.7]	0.5633	393.5 [-62.2, 849.1]	746.5 [290.9, 1201.2]	**0.0002**

Abbreviations: CI: 95% confidence interval from linear mixed effects model. ***Statistical significance:*** Significant p-values (<0.05) of linear mixed effects model are shown in bold font.

Table S4. Spearman correlations (Rs values) between cytokines and behavioral tests.

Cytokine	Neuroscore 2 d		Neuroscore 7 d		Neuroscore 14 d		Neuroscore 28 d		Beam-walking 2 d		Beam-walking 7 d		Beam-walking 14 d		Beam-walking 28 d	
	Vehicle-TBI	Clomi-TBI	Vehicle-TBI	Clomi-TBI	Vehicle-TBI	Clomi-TBI	Vehicle-TBI	Clomi-TBI	Vehicle-TBI	Clomi-TBI	Vehicle-TBI	Clomi-TBI	Vehicle-TBI	Clomi-TBI	Vehicle-TBI	Clomi-TBI
**Cytokines at 7 d**																
GM-CSF	-0.777*	-0.056	-0.286	-0.078	-0.464	0.156	-0.19	-0.263	-0.202	-0.149	-0.163	-0.203*	-0.144	0.147	-0.069	0.138
IL-1α	-0.675*	-0.037	-0.198	-0.096	-0.396	0.087	-0.215	-0.287	-0.104	-0.229	0.053	-0.147	0.038	0.239	0.002	0.106
IL-1β	-0.688*	-0.028	-0.248	-0.092	-0.418	0.142	-0.300	-0.225	-0.104	-0.182	0.031	-0.088	0.015	0.175	-0.037	0.074
IL-4	-0.689*	-0.107	-0.203	-0.092	-0.450	0.101	-0.179	-0.291	-0.187	-0.135	-0.067	-0.221	-0.058	0.179	0.121	0.156
IL-5	-0.637*	-0.149	-0.279	-0.183	-0.437	0.325	-0.347	-0.263	-0.091	-0.28	0.064	-0.065	0.024	0.290	-0.095	0.120
IL-6	-0.559*	0.000	-0.119	-0.073	-0.333	0.265	-0.227	-0.277	0.031	0.159	0.114	0.083	0.171	0.428	0.141	0.455
IL-10	-0.664*	0.033	-0.181	-0.082	-0.367	0.101	-0.255	-0.277	-0.02	-0.163	0.067	-0.106	0.064	0.221	0.019	0.143
IL-12p70	-0.723*	-0.033	-0.235	-0.064	-0.457	0.124	-0.171	-0.244	-0.149	-0.14	-0.089	-0.134	-0.064	0.175	-0.113	0.115
IL-13	-0.530*	-0.014	-0.036	-0.105	-0.307	0.256	-0.118	-0.348	0.049	0.201	0.126	0.152	0.222	0.414	0.290	0.469
IL-2	-0.611*	0.093	-0.120	-0.055	-0.422	0.284	-0.157	-0.263	-0.115	0.023	-0.002	-0.032	0.076	0.147	0.171	0.271
IFNy	-0.594*	0.000	-0.023	-0.151	-0.228	0.229	-0.141	-0.380	0.089	0.079	0.087	0.069	0.169	0.345	0.124	0.391
TNFα	-0.664*	-0.079	-0.176	-0.142	-0.363	0.142	-0.233	-0.362	-0.02	-0.093	0.078	-0.152	0.071	0.198	-0.017	0.221
**Cytokines at 14 d**																
GM-CSF	0.016	0.362	0.168	0.762*	0.218	0.337	-0.044	0.488	0.291	0.786	0.244	0.712*	0.429	0.441	0.163	0.483
IL-1α	0.132	0.289	0.232	0.746*	0.244	0.471	-0.038	0.484	0.328	0.751	0.318	0.668*	0.476	0.418	0.200	0.497
IL-1β	0.091	0.312	0.206	0.728*	0.244	0.421	-0.042	0.432	0.319	0.76	0.278	0.765*	0.443	0.395	0.195	0.460
IL-4	0.070	0.383	0.21	0.745*	0.270	0.420	-0.035	0.412	0.337	0.888	0.218	0.686*	0.394	0.408	0.145	0.551
IL-5	0.077	0.177	0.147	0.668*	0.223	0.485	-0.046	0.395	0.271	0.662	0.296	0.654*	0.478	0.474	0.162	0.487
IL-6	0.138	0.229	0.166	0.677*	0.273	0.411	-0.106	0.374	0.304	0.827	0.346	0.764*	0.467	0.468	0.139	0.541
IL-10	0.088	0.247	0.221	0.682*	0.235	0.499	-0.032	0.404	0.337	0.728	0.307	0.696*	0.477	0.455	0.204	0.524
IL-12p70	0.068	0.323	0.220	0.773*	0.227	0.415	0.010	0.501	0.341	0.804	0.249	0.686*	0.445	0.436	0.199	0.509
IL-13	0.134	0.428	0.199	0.637*	0.264	0.189	-0.091	0.275	0.315	0.880	0.386	0.730*	0.487	0.594*	0.238	0.677*
IL-2	0.111	0.514	0.195	0.755*	0.253	0.392	-0.104	0.407	0.273	0.893	0.256	0.760*	0.376	0.454*	0.212	0.620*

IFNy	0.014	0.378	0.113	0.773*	0.159	0.307	-0.173	0.334	0.240	0.886	0.251	0.705*	0.363	0.533*	0.148	0.658
TNFα	0.059	0.323	0.156	0.755*	0.216	0.438	-0.091	0.483	0.277	0.837	0.244	0.755*	0.403	0.399	0.160	0.495
**Cytokines at 28 d**																
GM-CSF	0.115	0.438	-0.339	0.297	-0.298	0.514	-0.361	0.326	-0.226	0.189	0.387	0.438	0.231	-0.133	0.037	0.027
IL-1α	0.000	0.212	-0.117	0.330	-0.321	0.686*	-0.267	0.216	-0.171	0.456	0.350	0.627*	0.203	-0.026	0.240	0.256
IL-1β	0.188	0.042	0.003	0.383	-0.099	0.619*	-0.034	0.247	-0.025	0.395	0.521	0.558	0.396	-0.241	0.140	0.032
IL-4	0.165	0.269	0.002	0.384	-0.174	0.542	-0.079	0.261	0.023	0.479	0.559*	0.735*	0.398	0.029	0.243	0.198
IL-5	0.164	0.235	-0.024	0.314	-0.089	0.688*	-0.119	0.209	-0.039	0.409	0.507	0.600	0.389	-0.07	0.121	0.218
IL-6	0.225	0.269	-0.127	0.384	-0.048	0.542	-0.334	0.261	-0.073	0.479	0.417	0.735	0.308	0.029	0.088	0.198
IL-10	0.238	0.272	0.052	0.330	-0.022	0.499	-0.078	0.132	0.044	0.396	0.567*	0.698*	0.440	0.009	0.174	0.198
IL-12p70	0.169	0.188	0.066	0.314	-0.126	0.600	-0.012	0.128	0.029	0.400	0.537	0.656*	0.406	-0.056	0.376	0.195
IL-13	0.141	0.239	-0.131	0.462	-0.214	0.444	-0.253	0.346	-0.059	0.425	0.444	0.626*	0.320	-0.118	0.180	0.043
IL-2	0.185	0.200	-0.086	0.382	-0.166	0.429	-0.227	0.185	-0.024	0.387	0.470	0.698*	0.340	-0.080	0.198	0.066
IFNy	0.054	0.269	-0.130	0.384	-0.255	0.542	-0.281	0.261	-0.148	0.479	0.443	0.735*	0.266	0.029	0.222	0.198
TNFα	0.207	0.301	-0.119	0.281	-0.014	0.454	-0.175	0.269	-0.144	0.430	0.454	0.705*	0.304	-0.068	0.011	0.111

Statistical significance: Significant Rs values: *, <0.05.

**Table S5.** Feature sets used in machine learning analysis.	
**Feature set**	**Variables**
Weight	Body weight measured at baseline, on the day of TBI induction, and at 1-7 d, 9 d, 11 d, 14 d, and 17 d after TBI
Temperature	Temperature measured at baseline and at 1-7 d, 9 d,11 d,14 d, and 17 d after TBI
Neuroscore	Total neuroscore at 2 d, 7 d, 14 d, and 28 d after TBI
Beamwalk	Beamwalk score at 2 d, 7 d, 14 d, and 28 d after TBI
MWM	Probe latencies at days 3 and 5
Cytokines	Cytokines (IL-1α, IL-1β , IL-2, IL-4, IL-5, IL-6, IL-10, IL-12 p70, IL-13, GM-CSF, INFγ and TNFα) at 7 d, 14 d, and 28 d after TBI
TBI	Duration of apnea, duration of post-impact seizure-like behavior, and impact pressure

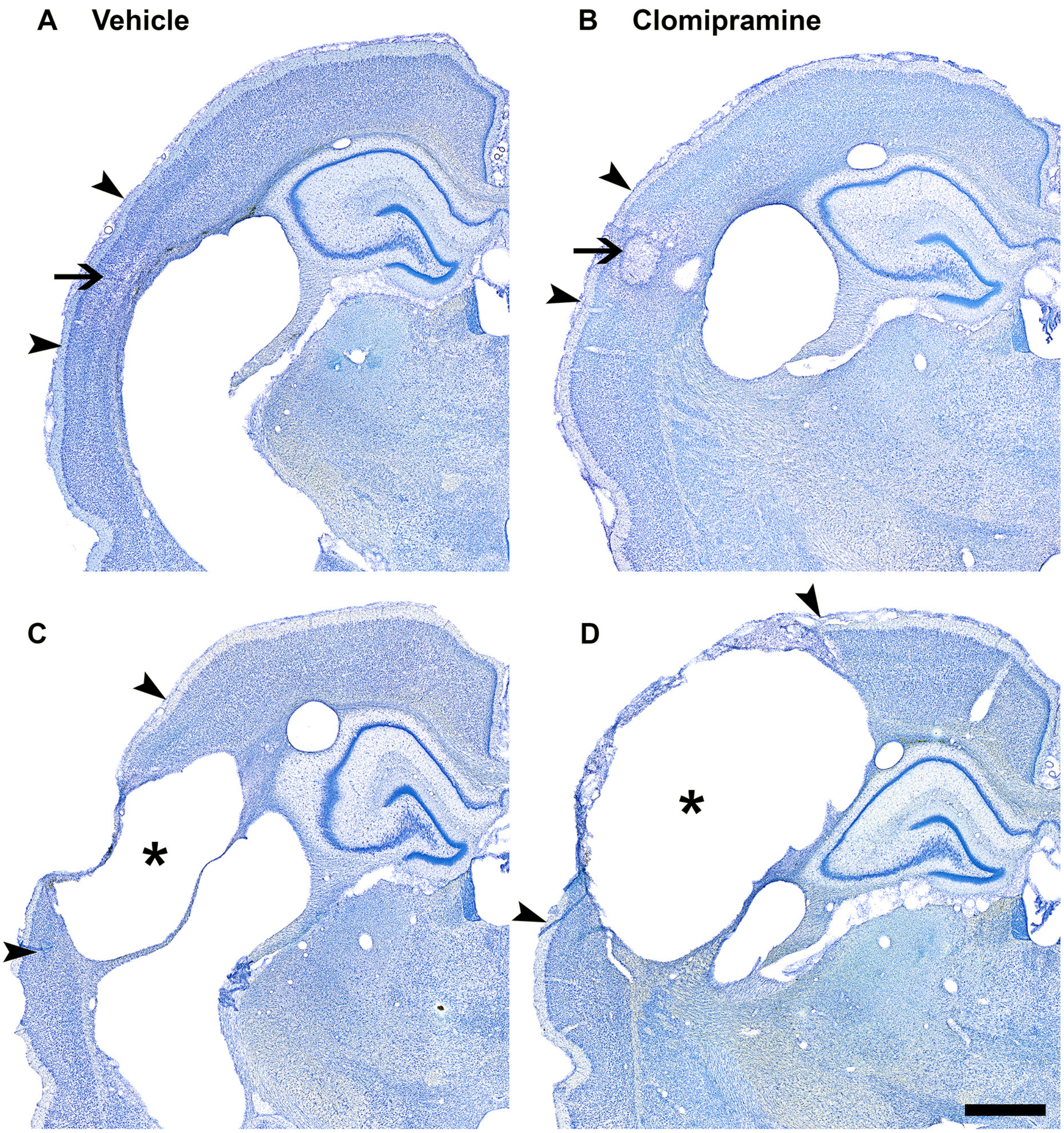

Figure S1. Thionin-stained coronal brain sections of the vehicle (panels A, C) and clomipramine –treated (panels B, D) rats at 28 days post-TBI, showing a comparable range of lesion severities. **(A)** A vehicle treated rat with a small cortical lesion. **(B)** A clomipramine-treated rat with small cortical lesion. **(C)** A vehicle treated rat with large cortical lesion. **(D)** A clomipramine-treated rat with a large cortical lesion. Arrowheads in panels A-D indicate the dorsoventral extent of the cortical lesion. Black arrows in panels A and B indicate the epicenter of the cortical lesion in A and B. Stars in C and D indicate the lesion cavity. Scale bar equals 1 mm (all panels).
